# The economics of healthcare access: a scoping review on the economic impact of healthcare access for vulnerable urban populations in low- and middle-income countries

**DOI:** 10.1186/s12939-022-01804-3

**Published:** 2022-12-31

**Authors:** Noemia Teixeira de Siqueira Filha, Jinshuo Li, Penelope A. Phillips-Howard, Zahidul Quayyum, Eliud Kibuchi, Md Imran Hossain Mithu, Aishwarya Vidyasagaran, Varun Sai, Farzana Manzoor, Robinson Karuga, Abdul Awal, Ivy Chumo, Vinodkumar Rao, Blessing Mberu, John Smith, Samuel Saidu, Rachel Tolhurst, Sumit Mazumdar, Laura Rosu, Surekha Garimella, Helen Elsey

**Affiliations:** 1 Department of Health Sciences, University of York, York, UK; 2 Liverpool School of Tropical Medicine, Department of Clinical Sciences, Liverpool, UK; 3 James P Grant School of Public Health, BRAC University, Dhaka, Bangladesh; 4 MRC/CSO Social &, University of Glasgow, Public Health Sciences Unit, Glasgow, UK; 5 The George Institute for Global Health, New Delhi, India; 6 LVCT Health, Nairobi, Kenya; 7 African Population and Health Research Center, Nairobi, Kenya; 8 The Society for Promotion of Area Resource Centres, Mumbai, India; 9 COMAHS: University of Sierra Leone, Freetown, Sierra Leone; 10 Liverpool School of Tropical Medicine, Department of International Public Health, Liverpool, UK; 11 University of York, Centre for Health Economics, York, UK

**Keywords:** Scoping review, Informal settlements, Slum, Costs, Catastrophic health expenditure, Low, Middle-income countries, Health economics

## Abstract

**Background:**

The growing urban population imposes additional challenges for health systems in low- and middle-income countries (LMICs). We explored the economic burden and inequities in healthcare utilisation across slum, non-slum and levels of wealth among urban residents in LMICs.

**Methods:**

This scoping review presents a narrative synthesis and descriptive analysis of studies conducted in urban areas of LMICs. We categorised studies as conducted only in slums, city-wide studies with measures of wealth and conducted in both slums and non-slums settlements. We estimated the mean costs of accessing healthcare, the incidence of catastrophic health expenditures (CHE) and the progressiveness and equity of health expenditures. The definitions of slums used in the studies were mapped against the 2018 UN-Habitat definition. We developed an evidence map to identify research gaps on the economics of healthcare access in LMICs.

**Results:**

We identified 64 studies for inclusion, the majority of which were from South-East Asia (59%) and classified as city-wide (58%). We found severe economic burden across health conditions, wealth quintiles and study types. Compared with city-wide studies, slum studies reported higher direct costs of accessing health care for acute conditions and lower costs for chronic and unspecified health conditions. Healthcare expenditures for chronic conditions were highest amongst the richest wealth quintiles for slum studies and more equally distributed across all wealth quintiles for city-wide studies. The incidence of CHE was similar across all wealth quintiles in slum studies and concentrated among the poorest residents in city-wide studies. None of the definitions of slums used covered all characteristics proposed by UN-Habitat. The evidence map showed that city-wide studies, studies conducted in India and studies on unspecified health conditions dominated the current evidence on the economics of healthcare access. Most of the evidence was classified as poor quality.

**Conclusions:**

Our findings indicated that city-wide and slums residents have different expenditure patterns when accessing healthcare. Financial protection schemes must consider the complexity of healthcare provision in the urban context. Further research is needed to understand the causes of inequities in healthcare expenditure in rapidly expanding and evolving cities in LMICs.

**Supplementary Information:**

The online version contains supplementary material available at 10.1186/s12939-022-01804-3.

## Introduction

Cities in low- and middle-income countries (LMICs) are characterised by large and growing urban populations [1]. The United Nations estimates that 2.9 billion people were living in cities in 2018 [2]. This growth has been coupled with worsening income inequalities, with the gap between rich and poor widening consistently across LMIC contexts since the 1980s [2]. Urban poverty, while characterised by common domains, takes many different forms [3]. The unacceptable poor living conditions of informal settlements provide a blatant illustration of urban poverty, yet poverty is spread throughout cities with individual or smaller clusters of poor households within better off neighbourhoods. Understanding how these different forms of urban poverty may influence healthcare utilisation and health outcomes is complex and hampered by a lack of granularity of within-city population and health data [4–7].

Many researchers have focused on informal settlements or slums as an accessible way to understand urban poverty as well as in response to their blatant and visibly unacceptable conditions. UN-Habitat estimates that 38% and 54% of the urban population in South Asia and sub-Saharan Africa respectively were living in slum settlements in 2018 [8]. While the proportion of slum dwellers within LMIC cities have reduced, absolute numbers have increased over the last decade [9]. The number, size and morphology of these slums vary considerably across countries and cities [10]. The transient nature of urban–rural migration with people continually arriving and moving around the city, with some leaving the city seasonally or as their livelihoods demand [11] adds further complexity to understanding the relationship between urban poverty and healthcare access [12].

In addition to the complexities of urban demographics, health systems and determinants within cities are complex, dynamic and often significantly different from rural areas. Urbanisation itself has been identified as a determinant of health [13]. The rapid and uncontrolled urbanisation that has characterised LMICs and the resultant disparities in economic conditions has led to widening health and wellbeing inequalities [14, 15]. Changes in living conditions and health-seeking behaviours, coupled with insufficient access to quality healthcare undermine the opportunities for city residents, particularly slum-dwellers, new migrants and low-income households whose unstable and informal working lives present further challenges to access appropriate quality care and keeping healthy [16, 17]. The limited provision of public primary care, the number, range and complexity of private providers [18] and the wide variations in the quality of healthcare provision in cities present significant challenges to effective health-seeking behaviour[19]. The diversity of private providers is well documented and ranges from licenced and unlicensed independent practitioners, non-government organisation (NGO) providers, corporate hospital chains and itinerant medicine sellers [18]. Evidence suggests that the urban poor predominantly use unlicensed practitioners and/or poor-quality health services [20]. The lack of good quality public services and reliance on the private sector may result in high costs to access healthcare. The poorest are also more likely to incur catastrophic health expenditures (CHE), here defined as the total amount of healthcare expenditure exceeding a pre-determined threshold of the household income or capacity to pay.

We undertook this scoping review to assess the economic impact of healthcare access across different poor urban populations within cities and determine the progressiveness and inequities in healthcare expenditures in cities in LMICs.

## Methods

### Overview

This review was developed as part of the ARISE research consortium which aims to enhance accountability and improve the equitable health and wellbeing of marginalised populations living in slums in LMICs [21]. This is a collaborative study developed by representatives from the ARISE partner organisations in the UK, India, Bangladesh, Sierra Leone and Kenya. The research question and protocol [22] for the scoping review were developed in close discussion with ARISE partners including those who work directly with communities in informal settlements in India, Bangladesh, Sierra Leone and Kenya. This helped to shape the focus of the review and to ensure the findings are relevant at a city level.

The review was conducted following a framework proposed by Arksey and O’Malley (2005) [23] and Levac et al. (2010) [24], with five stages of development (1) identifying the research question, (2) identifying relevant studies, (3) study selection, (4) charting the data, and (5) collating, summarizing and reporting the results. We reported this scoping study according to the PRISMA extension for scoping reviews (PRISMA-ScR) Checklist [25] and Synthesis Without Meta-analysis (SWiM) reporting guideline [26]. The protocol of this review has been registered at the Research Registry platform (https://www.researchregistry.com/, ID: reviewregistry947), and published [22].

The review team members had diverse backgrounds and a range of experience in systematic and scoping reviews. Therefore, to promote capacity building and skills development, core principles within the ARISE hub, the review team was divided into three groups, with members allocated according to their skills and time available to contribute. The *core team* was comprised of researchers with experience in conducting systematic reviews and/or data analysis. The *new reviewer* team included junior researchers with little experience in review methods. The *core* and *new reviewer* teams worked through a mentorship scheme and collaborated in all phases of the review. The third team was the *advisory group* and included senior researchers with expertise in quantitative data analysis and urban health. They contributed to the interpretation of data and developing policy briefs to communicate the review results to a wider audience in the countries where the ARISE hub primary research is taking place.

### Research questions

This review aimed to answer the following research questions:

1. Does the definition of slums follow the criteria proposed by the UN-Habitat (2018)?
2. What is the mean cost of accessing healthcare for urban populations in LMICs?
3. What is the progressiveness and equity pattern of health expenditures across the urban population in LMICs?
4. What is the prevalence of CHE incurred by the urban population in LMICs?
5. What are the evidence gaps in research addressing the economics of healthcare access in LMICs?

### Search strategy

The search strategy used the following key terms and concepts: (slum dwellers OR slums OR informal settlements) AND (urban areas) AND (healthcare costs) AND (low-middle income countries) (supplementary file). Retrieval was limited to publications within the last 10 years (2010–2020) as changes in urban health have mainly occurred over the past decade due to the implementation of the Millennium Development Goals [27]. We searched the literature in MEDLINE (Ovid), Embase (Ovid), EconLit (Ovid), Science Citation Index (Web of Science), Social Science Citation Index (Web of Science), Global Index Medicus, and Proquest Dissertations and Theses (A&I) on June 29^th^ 2020. EndNote was used for reference management and duplicate removal. Non-open access articles were requested to the British Library by the interlending service. No attempts were made to contact the authors.

### Study selection, eligibility and exclusions

We included cost studies estimating direct and indirect costs and/or CHE incurred during the search for healthcare by slum and city-wide urban residents of LMICs. Studies reporting data from both rural and urban areas and urban studies not mentioning the inclusion of slum-dwellers in the sample were included if results were reported disaggregated by wealth quintile. We included the publication with the most complete report of costs (direct medical, non-medical and indirect) and sample size per wealth quintile when multiple publications reported results from the same study population. Peer review articles, theses, dissertations, working papers and reports in English, French, Spanish, Chinese and Portuguese were included in this review (Table S1 and Figure S1).

### Screening, data charting and quality assessment

The review team was separated into screening groups for the screening of titles and abstracts. Each group included at least one reviewer from the *core team*. Reviewers independently screened a set of 500 titles and abstracts each. The full-text screening followed the same approach and was independently performed by six pairs of reviewers (NTSF and AA; PAPH and FM; JL and IHM; ZQ and RK; HE and AV; EK and VS), with one reviewer from the *core team* and the second from the *new reviewers team*. The screening was performed using Rayyan web tool (http://rayyan.qcri.org) [28]. Discordances were discussed and resolved during weekly online meetings.

Data extraction and quality assessment were conducted independently by the same pairs of reviewers using COVIDENCE web tool [29] with consensus reached by a third reviewer. We developed a data charting form to extract data from each study (e.g. study design, methods, cost components and estimates) (Table S2). While scoping reviews do not normally require quality assessment, as we wished to analyse study results to compute mean costs and concentration curves and indices we felt it important to assess study quality to inform our level of confidence in our synthesised results. Therefore, we decided to perform the quality assessment to support the interpretation of results and build the evidence map. As different study designs (e.g. longitudinal and cross-sectional surveys) were reported, we extracted relevant sections from three checklists and combined them to examine the quality of the studies: (1) Tool to Estimate Patient’s Costs (TBCA) [30], recommended for studies evaluating patient costs, (2) Consolidated Health Economic Evaluation Reporting Standards (CHEERS) [31], recommended for economic evaluations, and (3) Assessment Tool for Observational Cohort and Cross-Sectional Studies from the National Heart, Lung and Blood Institute (NIH) [32]. We added two more questions in our quality assessment tool that were judged as important to complement the quality appraisal of the included studies: *Was the method used for estimating catastrophic health expenditures explained? Was an appropriate methodology applied to calculate income level described and adequate?* (Table S3). We classified the studies into three categories according to the proportion of agreement with the quality criteria (1) good: quality score > 85% of agreement, (2) fair: quality score between 75 and 84% of agreement, (3) poor: quality score < 75% of agreement.

### Data synthesis, presentation and analysis

We tabulated our findings as a narrative synthesis and present quantitative data in tables. We also performed descriptive analysis to estimate the economic burden of healthcare access (mean costs and CHE) and the progressiveness and equity of health expenditures.

Studies reporting data from multiple slums, years, countries, or health sectors (e.g. public and private) were analysed as separate observations. We also considered as separate observations slum/non-slum studies providing disaggregated costs by study site.

Following our initial scoping of the urban populations sampled within the included studies, we classified studies into (1) slum studies: studies clearly stating the study area as a slum, informal settlement or relocation colonies according to the study authors’ definition; (2) city-wide studies: studies in urban areas presenting the economic burden by wealth quintiles but no statement of slums, informal settlements, or relocation colonies as the study area; (3) slum/non-slum studies: studies clearly stating the study area as slum settlements and other city areas (non-slum).

### Question 1: Does the definition of slums follow the criteria proposed by the UN-Habitat (2018)?

The slum definitions used by the study authors were mapped against the three characteristics of informal settlements put forward by UN-Habitat (2018): (1) inhabitants have no security of tenure vis-à-vis the land or dwellings they inhabit, with modalities ranging from squatting to informal rental housing; (2) neighbourhoods usually lack, or are cut off from basic services and formal city infrastructure; (3) housing may not comply with current planning and building regulations, situated in geographically and environmentally hazardous areas, and may lack a municipal permit [33].

We categorised the health conditions as acute, chronic and unspecified conditions. Unspecified conditions refer specifically to the studies reporting general healthcare spending regardless of the reason. Table S4 shows the diseases and other conditions included in these categories.

### Question 2: What is the mean cost of accessing healthcare for urban populations in LMICs?

Costs were classified as direct costs (medical and non-medical out-of-pocket expenditure on medications, diagnostics tests, hospital or ambulatory fees, transportation, and others), indirect costs (income/productivity loss) and total costs (direct plus indirect costs). We estimated the average of means with standard deviation (SD), and the median of means with interquartile range (IQR) of comparable studies (i.e. same health condition, type of costs, time horizon and cost unit). Costs were presented by study type, health condition and wealth quintile. We reported the cost of the last illness episode or care for acute conditions and costs incurred during the one-year time horizon for chronic and unspecified health conditions.

All costs were reported in International Dollars (I$) in 2020 prices to allow comparability across the studies. We first inflated costs reported in the local currency to 2020 prices by using the yearly inflation rates reported by the International Monetary Fund [34]. Costs reported in US Dollars were converted to the local currency by using the exchange rates reported in the study and then inflated to 2020 prices. To obtain costs in I$, inflated costs were divided by the annual purchase power parity conversion factor reported by the World Bank for each country, 2020 values [35].

### Question 3: What is the progressiveness and equity pattern of health expenditures across the urban population in LMICs?

We computed concentration curves and concentration indices for slum and city-wide studies providing cost and sample size per wealth quintile. We aimed to identify the progressiveness and equity of health expenditures. The concentration curve was used to display the share of health (cumulative direct costs of chronic conditions) by the cumulative population ranked in wealth quintiles (from poorest to richest). Analysis of the expenditure patterns was based on the line of equality, a 45-degree line where everyone has the same healthcare expenditure. Concentration curves placed below the line of equality indicate that costs were concentrated among the richest, whilst curves placed above the equality line indicate that costs were concentrated among the poorest. The concentration indices, twice the area between the concentration curve and the line of equality, were calculated to measure the magnitude of inequities in healthcare expenditures. The indices vary from -1 to 1, with negative values indicating healthcare expenditures concentrated among the poorest and positive values indicating expenditures concentrated among the richest wealth quintiles [36, 37].

The concentration indices were calculated by using the formula:

$$C=(p1L2\;-\;p2L1)\;+(\;p2L3\;-\;p3L2)\;+...+(\;pT\;-1LT\;-\;pT\;LT-1)$$

*Where p*_*t*_ *is the cumulative percentage of the sample ranked by economic status in group t (cumulative population ranked in wealth quintiles), and L*_*t*_ *is the corresponding concentration curve ordinate (cumulative healthcare expenditure).*

### Question 4: What is the prevalence of CHE incurred by the urban population in LMICs?

We computed the average incidence of CHE for chronic and unspecified conditions in Box and Whisker charts by study type and wealth quintile applying 10% and 15% thresholds.

### Question 5: What are the evidence gaps in research addressing the economics of healthcare access in LMICs?

We developed an evidence map [38] to analyse research gaps and the strength of evidence. We plotted bubble charts to present the body of evidence in slum, city-wide and slum/non-slum studies. We analysed the number of publications (total and reporting CHE), the most frequent health conditions (unspecified, obstetric/neonatal care, chronic conditions and others combined), and countries (India, Bangladesh, China, and others combined). The size of the bubbles indicates the total sample size, the X axis indicates the total number of studies and the Y axis indicate the average strength of evidence.

## Results

**Fig. 1:**
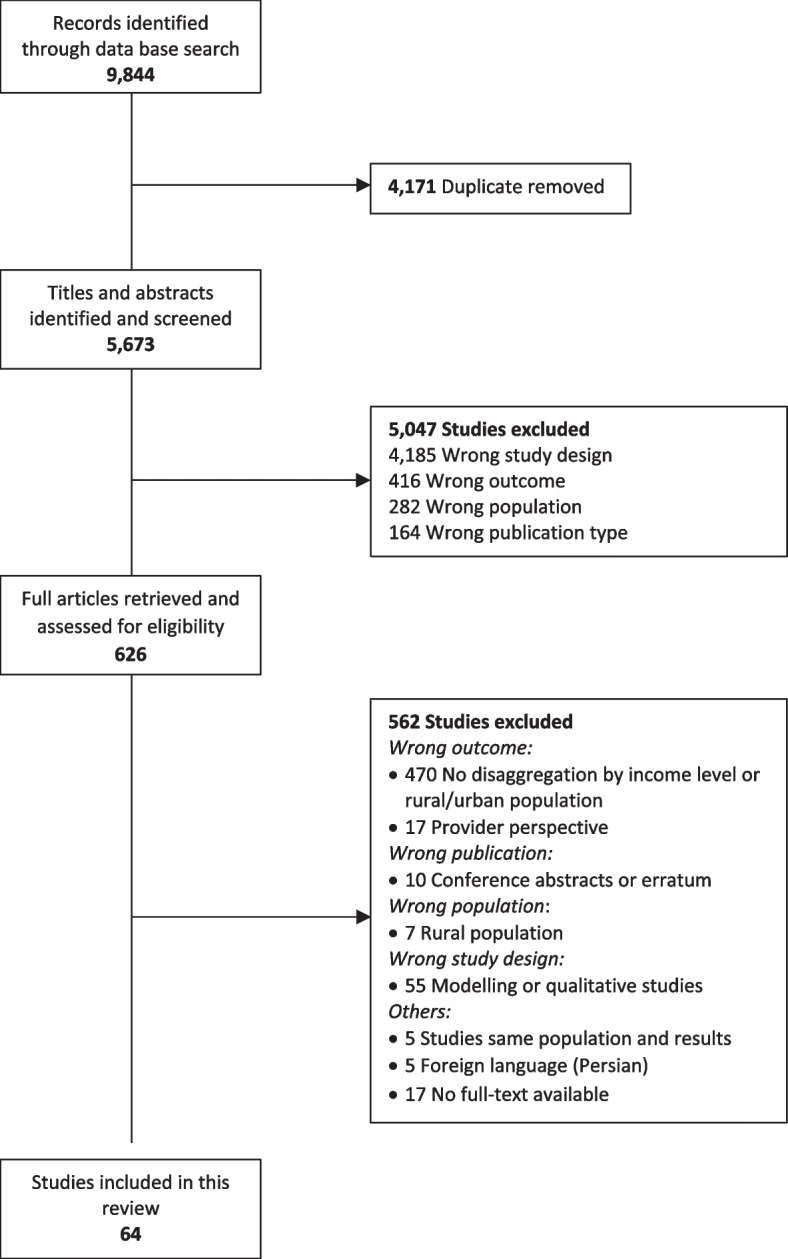
Prisma Chart showing references retrieved at different stages of the search

Of 5,673 unique records identified, 64 were included in our review, most of which were published after 2014 (40, 63%). The main reason for exclusion after the full-text screening was the lack of disaggregated data by income level and/or rural and urban areas which led to the exclusion of 470 studies (Fig. 1).

Studies were mainly from the South-East Asian Region (38, 59%), followed by the Western Pacific Region and the African Region (8, 12%, each), the American Region (7, 11%) and the Eastern Mediterranean Region (3, 5%). India had the highest number of studies retrieved (27, 42%). More than half of the articles (37, 58%) were classified as city-wide studies presenting analysis across wealth categories, 23 as slum studies (36%) and slum/non-slum areas (6%). Studies addressed a variety of health conditions such as obstetric and/or neonatal care, tuberculosis, injuries, and other communicable and non-communicable diseases (Table 1).

**Table 1:** Characteristics of included studies

**Reference**	**Country and geographical area**	**Study design**	**Study population**	**Health interest**	**Type of insurance coverage**	**Sampling method, sample size**	**disaggregate income levels**	**Method applied to calculate household income and SES**
Das, 2010 [39]	• India • Urban/peri-urban • Slum	Longitudinal survey	Women and new-borns	Obstetric and/or neonatal care	NR	• Purposive • 10,754 births • Institutional Deliveries: 9,046 • Home deliveries: 1708	NR	Asset score
Barros, 2011 [40]	• Brazil • Urban/peri- • City-wide	Cross-sectional	General population	Unspecified health-care	NR	• Systematic and random • 37,830 households	Wealth quintile	National Economic Indicator (asset index)
Lopera, 2011 [41]	• Colombia • Urban/peri-urban • City-wide	Cross-sectional	General population	HIV	Contributive plan, subsidized plan public and private	• Convenience • 540 individuals	Wealth sextile	NR
Garcia, 2012 [42]	• Brazil • Urban and rural • City-wide	Cross-sectional - National household survey	General population	Unspecified health-care	Private	• 31,253 households • 1995-1996: 16,060 households • 2002-2003: 6,594 households • 2008-2009: 8599 HH	Wealth quintile	NR
Saini, 2012 [43]	• India • Urban/peri-urban • Slum	Cross-sectional	Women and new-borns	Obstetric and/or neonatal care	NR	• Systematic • 360 households	NR	NR
Skordis-Worrall, 2012 [44]	• India • Urban/peri-urban • Slum	Cross-sectional	Women and new-borns	Obstetric and/or neonatal care	NR	• Purposive • 1,204 post-partum women	Wealth quintile	Principal Component Analysis (PCA): asset based index
Bhojani, 2012 [45]	• India • Urban/peri-urban • Slum	Cross-sectional - National household survey	General population	Chronic illness	NR	• Purposive • 9,299 households • 3,202 with chronic diseases • 6,097 without chronic diseases	Wealth quintile	Per capita income
Kumar, 2012 [46]	• India • Urban/peri-urban • City-wide	Longitudinal survey	General population	Injuries	NR	• Convenience • 756 individuals • Arrived alive: 723 • Arrived dead: 34	Wealth quartile	Monthly household income
Weraphong, 2013 [47]	• Thailand • Urban/peri-urban • Slum/non-slum	Cross-sectional household survey	General population	Unspecified health-care	Social/national health insurance	• Multistage random • 406 sampled households	Wealth decile	Poverty line
Sarker, 2013 [48]	• Bangladesh • Urban/peri-urban • City-wide	Longitudinal survey	General population	Acute illness	NR	• Convenience • 394 individuals and 400 households	Wealth quintile	Equivalence scale
Sakdapolrak, 2013 [49]	• India • Urban/peri-urban • Slum	Mixed method study	General population	Unspecified health-care	NR	• Random • 1st panel: 1,041 individuals/219 households • 2nd panel: 100 households	Wealth quartile	NR
Misra, 2013 [50]	• India • Urban/peri-urban • Slum	Longitudinal survey	General population	Unspecified health-care	Social/national health insurance	• Multistage cluster • 400 households	Wealth quintile	Monthly household consumption expenditure
Rahman, 2013 [51]	• Bangladesh • Urban/peri-urban • City-wide	Cross-sectional household survey	General population	Chronic illness	NR	• Multistage cluster • 1,593 households	Wealth quintile	Household consumption expenditure
Patel, 2013 [52]	• India • Urban/peri-urban • Slum	Longitudinal survey	General population	Acute illness	NR	• Random • 400 households	NR	NR
Seeberg, 2014 [53]	• India, Indonesia, Thailand • Urban/peri-urban • Slum/non-slum	Longitudinal survey	General population	Unspecified health-care	Social/national health insurance	• Purposive • 11,418 individuals in 2,608 • India: 6,757 individuals in 1,394 households • Indonesia: 3,473 individuals in 863 households • Thailand: 1,188 in 351 households	Wealth decile	Median monthly household income
Ilesanmi, 2014 [54]	• Nigeria • Urban/peri-urban • City-wide	Cross-sectional	General population	Unspecified health-care	Social/national health insurance	• Multistage • 14 households	Wealth quintile	Principal Component Analysis (PCA): assest based index
Wingfield, 2014 [55]	• Peru • Urban/peri-urban • Slum	Longitudinal survey	General population	Tuberculosis	NR	• Convenience • 876 tuberculosis patients and 487 health controls • TB patients 876, • non-MDR : 783 • MDR: 93	Wealth tertile	Composite household poverty index
Chenge, 2014 [56]	• DRC • Urban/peri-urban • Slum	Cross-sectional household survey	General population	Unspecified health-care	NR	• Multistage cluster and random • 251 households	Wealth quintile	Index calculated on the basis of weighted categories
Navneet, 2014 [57]	• India • Urban/peri-urban • Slum	Cross-sectional	General population	Unspecified health-care	NR	• Random • 1,121 individuals in 132 households • MaIe: 579 • Female: 542	NR	NR
Saito, 2014 [58]	• Nepal • Urban/peri-urban • City-wide	Cross-sectional	General population	Unspecified health-care	NR	• Systematic • 1997 households	Wealth quintile	Household expenditure
Rehman, 2014 [59]	• Pakistan • Urban/peri-urban • Slum	Cross-sectional	Children	Unspecified health-care	NR	• Multistage • 252 children in 252 households	NR	NR
Prabhakaran, 2014 [60]	• India • Urban/peri-urban • Slum	Cross-sectional household survey	General population	Unspecified health-care	NR	• Stratified • 335 individuals	NR	Kuppaswamy classification
Tripathi, 2014 [61]	• India • Urban/peri-urban • Slum	Cross-sectional household survey	Women and new-borns	Obstetric and/or neonatal care	NR	• Purposive • 425 womenJanani Shishu Suraksha Karyakaram (JSSK) • Pre-JSSK: 233 women • Post-JSSK: 192 women	NR	Per capita income
Chandra, 2014 [62]	• India • Urban/peri-urban • City-wide	Cross-sectional	General population	Chronic illness	NR	• Purposive • 219 patients • Males: 129 • Females: 90	Wealth quintile	Kuppaswamy classification
Joe, 2015 [63]	• India • Urban and rural • City-wide	Cross sectional	General population	Unspecified health-care	NR	• Stratified multistage • 73,000 households; • Rural: 47,000 • Urban: 26,000	Wealth quintile	Reported household consumption expenditure
da Silva, 2015 [64]	• Brazil • Urban/peri-urban • City-wide	Longitudinal survey	Children	Child health	Private	• 2,436 individuals	Wealth decile	Sum of incomes of all household members
Patle, 2015 [65]	• India • Urban/peri-urban • City-wide	Cross-sectional	Elderly	Unspecified health-care	NR	• Systematic random • 250 elderly persons • Male: 113 • Female: 137	Wealth decile	Poverty line
Putri, 2015 [66]	• Indonesia • Urban/peri-urban • City-wide	Cross-sectional	General population	Unspecified health-care	Social/national health insurance	• Multistage random and simple random • 918 households	Wealth quintile	Monthly income per capita
Thakare, 2015 [67]	• India • Urban/peri-urban • Slum	Cross-sectional	General population	Chronic illness	NR	• Random • 2,360 individuals in 447 households	NR	NR
Buigut, 2015 [68]	• Kenya • Urban/peri-urban • Slum	Cross-sectional household survey	General population	Unspecified health-care	NR	• Modified cluster sampling • 8,171 households	Wealth tertile	Household income
Davari, 2015 [69]	• Iran • Urban and rural • City-wide	Cross-sectional	General population	Unspecified health-care	NR	• Multistage random stratified and cluster • 1,716 households • 2004: 715 • 2011: 1,001	Wealth quintile	Poverty line
Loganathan, 2015 [70]	• Malaysia • Urban and rural • City-wide	Cross-sectional	Children	Acute illness	NR	• Convenience • 653 individuals • Urban: 467 • Rural: 333	Wealth quintile	NR
Khan, 2015 [71]	• Bangladesh • Urban/peri-urban • Slum	Longitudinal survey	General population	Unspecified health-care	NR	• Purposive and random • 614 households	NR	NR
Khaing, 2015 [72]	• Myanmar • Urban and rural • City-wide	Cross-sectional	General population	Unspecified health-care	NR	• Multistage random • 3,066 individuals in 700 households • Urban: 350 • Rural: 350	Wealth quintile	Annual household expenditure per capita
Wingfield, 2016 [73]	• Peru • Urban/peri-urban • Slum	Longitudinal survey	General population	Tuberculosis	NR	• Convenience • 282 individuals • Intervention: 135 • Healthy controls: 262	Wealth tertile	Principal Component Analysis (PCA)
Wang, 2016 [74]	• China • Urban and rural • City-wide	Cross-sectional household survey	General population	Unspecified health-care	NR	• Stratified, systematic and probability proportion size systematic • 37,605 individuals in 11,409 households • Urban: 2,391 households • Rural areas: 3,095 households	Wealth quintile	NR
Chen 2016 [75]	• China • Urban/peri-urban • City-wide	Cross-sectional	General population	Chronic illness	NR	• Convenience • 678 individuals • Beijing: 170 • Guangzhou: 175 • Shanghai: 168 • Chengdu: 165	Wealth quintile	Monthly household income per capita
Mishra, 2016 [76]	• India • Urban/peri-urban • Slum	Cross-sectional	Children	Child health	NR	• Convenience • 175 children • Boys: 94 • Girls: 81	Wealth tertile	Monthly household income
Kien, 2016 [77]	• Vietnam • Urban/peri-urban • Slum/non-slum	Cross-sectional household survey	General population	Chronic illness	Social/national health insurance	• Multistage cluster and random • 1,020 households • Slum areas: 492 • Non-slum areas: 528	Wealth quintile	Principal Component Analysis (PCA): asset based index
Khalid, 2016 [78]	• Pakistan • Urban and rural • City-wide	Cross-sectional - National household survey	General population	Unspecified health-care	Private	• Multistage • 91,404 households	Wealth decile	NR
Jeyashree, 2017 [79]	• India • Urban and rural • City-wide	Cross-sectional - National household survey	Elderly	Unspecified health-care	NR	• Stratified multistage • 27,245 elderly persons	Wealth quintile	Usual monthly per capita expenditure
Hendrix, 2017 [80]	• Malawi • Urban and rural • City-wide	Longitudinal survey	Children	Acute illness	NR	• Purposive • 514 individuals • Rural: 22 • Urban: 269	Wealth quintile	Self-reported monthly income
Sahu, 2017 [81]	• India • Urban/peri-urban • Slum	Cross-sectional	Women and new-borns	Obstetric and/or neonatal care	Social/national health insurance	• Random • 250 individuals	NR	NR
Khan, 2017 [82]	• Bangladesh • Urban and rural • City-wide	Cross-sectional - National household survey	General population	Unspecified health-care	NR	• Multistage stratified random • 12,240 households	Wealth quintile	Principal Component Analysis (PCA): asset based index
Xu, 2018 [83]	• China • Urban and rural • City-wide	Cross-sectional	General population	Chronic illness	Urban Employee Basic Medical Insurance, Urban Resident Basic Medical Insurance, and New Rural Cooperative Medical Insurance	• Multistage stratified random cluster • 9646 households • 2008: 1,942 • 2013: 7,704	Wealth quintile	Household expenditure
Sharma, 2018 [84]	• India • Urban/peri-urban • Slum	Cross-sectional	Women and new-borns	Obstetric and/or neonatal care	NR	• Multistage random • 184 households	Wealth decile	Kuppaswamy classification
Mukama, 2018 [85]	• Uganda • Urban/peri-urban • Slum	Cross-sectional	Children	Injuries	NR	• Purposive and multistage • 1,583 children	NR	NR
Ranjan, 2018 [86]	• India • Urban and rural • City-wide	Cross-sectional - National household survey	General population	Unspecified health-care	Social, private, employer-provided and special schemes such as Yashwasini (privately initiated and linked to cooperatives)	• Multistage stratified • 65,932 households and 57,456 hospitalization episodes • Rural: 36,480 households • Urban: 29452 households	Wealth quintile	Usual monthly per capita consumption expenditure (UMPCE)
Cascaes, 2018 [87]	• Brazil • Urban and rural • City-wide	Cross-sectional - National household survey	General population	Dental care	Private	• Multistage cluster and random • 2961 individuals	Wealth quintile	NR
Kusuma, 2018 [88]	• India • Urban/peri-urban • Slum	Cross-sectional	General population	Unspecified health-care	Government and employer	• Random clusters • 2,998 households	Wealth quintile	NR
Pandey, 2018 [89]	• India • Urban and rural • City-wide	Cross-sectional - National household survey	60 years or more and under 60 years	Unspecified health-care	NR	• 87,513 individuals	Wealth quintile	NR
Enweronu-Laryea, 2018 [90]	• Ghana • Urban/peri-urban • City-wide	Mixed method study	Women and new-borns	Obstetric and/or neonatal care	Social/national health insurance	• Random • 114 individuals • 56 mothers • 58 new-borns	Wealth tertile	Parental monthly income
Sepehri, 2019 [91]	• Vietnam • Urban and rural • City-wide	Cross-sectional - National household survey	General population	Injuries	NR	• Stratified cluster • 9,399 households • Rural: 6,615 • Urban: 2,781	Wealth quintile	Per capita consumption expenditure
Ntambue, 2019 [92]	• DRC • Urban/peri-urban • City-wide	Mixed method study	Women and new-borns	Obstetric and/or neonatal care	NR	• Convenience • 1,627 women	Wealth quintile	Principal Component Analysis (PCA)
Banerjee, 2019 [93]	• India • Urban/peri-urban • Slum	Cross-sectional	General population	Unspecified health-care	NR	• Stratified, purposive, random • 320 individuals	Wealth decile	Average monthly income
Attia-Konan, 2019 [94]	• Côte d'Ivoire • Urban and rural • City-wide	Cross-sectional	General population	Chronic illness	NR	• Stratified random • 47,635 individuals in 12,899 households	Wealth quintile	Household expenditure on consumables
Acharya, 2019 [95]	• Nepal • Urban/peri-urban • City-wide	Cross-sectional household survey	Elderly	Unspecified health-care	Government and private	• Multistage, stratified, cluster • 401 elderly persons	Wealth tertile	International Wealth Index (IWI), asset-based wealth indices
Bose, 2019 [96]	• India • Urban and rural • City-wide	Cross-sectional - National household survey	Elderly	Chronic illness	Government Funded and Employer Supported	• Stratified multistage and probability proportion to size with replacement • 333,104 individuals in 65,932 household	Wealth quartile	Organization for Economic Cooperation and Development (OECD) equivalence scale to construct the monthly per capita expenditure (MPCE) class from the household expenditure
Ma, 2019 [97]	• China • Urban and rural • City-wide	Longitudinal survey	General population	Unspecified health-care	NR	• Random • 51,880 individuals in 14,331 households • Urban: 25,354 • Rural: 26,526	Wealth quintile	NR
Leng, 2019 [98]	• China • Urban and rural • City-wide	Cross-sectional household survey	General population	Cancer	New Rural Cooperative Medical scheme, Medical Insurance for Urban Residents scheme, Medical Insurance for Urban Employees scheme	• Respondent-driven • 792 cancer patients • Urban: 195 • Rural: 597	Wealth quintile	Monthly household income
Muniyandi, 2020 [99]	• India • Urban/peri-urban • Slum/non-slum	Cross-sectional	General population	Tuberculosis	Direct Benefit Transfer and other support schemes	• Convenience • 384 individuals • Male: 256 • Female: 128	Wealth tertile	Household annual income
Poornima, 2020 [100]	• India • Urban/peri-urban • City-wide	Longitudinal survey	General population	Tuberculosis	NR	• Purposive • 214 individuals • Devangere: 79 • Belagavi: 90 • Bengaluru: 45	Wealth quintile	Modified BG Prasad classification
Adams, 2020 [101]	• Bangladesh • Urban/peri-urban • Slum	Cross-sectional household survey	General population	Chronic illness	NR	• Multistage cluster and purposive • 1,045 individuals • Sylhet: 509 • Tongi: 536	Wealth quintile	Progress of Poverty Index 1 (PPI)
Swetha, 2020 [102]	• India • Urban/peri-urban • City-wide	Longitudinal survey	General population	Unspecified health-care	NR	• Probability proportional to size sampling, random • 1,581 individuals in 350 households	Wealth quartile	NR

*NR* Not reported, *DRC* Democratic Republic of the Congo, *SES* Socio-economic status

### Definition of slums

Among 27 slum and slum/non-slum studies, 12 (44%) described the concept of slum and/or slum-dwellers. There was considerable variation in the definition of a slum, but none of the studies covered all characteristics proposed by the UN-Habitat. The danger of eviction was reported by one study (3.7%) [103], lack or poor access to basic services (water, electricity, sanitation) by six studies (22%) [39, 68, 104–107], and lack of planning and/or building regulation by eight studies (29.6%) [39, 68, 104, 106–109]. Additional elements were reported by nine studies (33.3%) [68, 103, 105–112] and included dense populations or shelters, discrimination of the inhabitants, low socio-economic status, high unemployment or employment in informal or low-skill jobs and inhabitants with poor health status (Table 2).

**Table 2:** Characteristics of slum dwellers according to the UN-Habitat reported by slum studies

Study	Security of tenure	Access to basic services and formal city infrastructure	Planning and building regulations	Additional elements
Das, 2010 [39]		A substantial proportion of households did not have metered electric supply, access to individual or communal piped water or individual toilet facilities	Situated on or beside hazardous locations like railway lines, garbage dumps and polluted bodies of water. Houses were of insubstantial construction	
Bhojani, 2012 [104]		Inadequate sanitary and drinking water facilities	Compact settlement of poorly built tenements	
Sakdapolrak, 2013 [103]	Danger of eviction		Not fully recognized and lack of basic infrastructure	Physical and spatial manifestation of urban poverty. Population suffers discriminatory and oppressive practice of unsociability
Patel, 2013 [105]		Poor access to sanitation and clean water due to non-existent or poorly developed basic infrastructure		Dense populations
Seeberg, 2014 [106]		Houses served by illegal electricity connections provided by a local contractor for a monthly fee. No regular water supply. Inhabitants dependent on two communal water sources, which provided water for two hours in a day and on water tankers that came once a day, and often in the summer once in two days	Houses built with concrete and tin roofs, but a majority with flammable roofs Single room hutments of approximately 50 m^2^ served as an all-purpose room generally demarcated into a sleeping area and a cooking area	Densely packed shelters
Buigut, 2015 [68]		Lack of access to piped water, poor environmental sanitation	Poor and unsafe dwelling structures	High unemployment and low incomes, low education levels, and high disease prevalence
Khan, 2015 [110]				Dwellers typically engaged in informal sector and low skill jobs occupations like hawking, trading, domestic work, rickshaw, brick breaking, construction
Kien, 2016 [109]			Located in narrow spaces and/or in polluted locations	A group of at least 30 households that are temporary and/or very old houses
Mukama, 2018 [111]				Informal and substandard housing and a high population density. It is primarily residential with small businesses such as kiosks and grocery stalls
Kusuma, 2019 [108]			Dwellings walled and roofed with tin/asbestos sheets. Questionable legality	Inhabited by the people, who migrated to work in the industries and factories long back and started living by establishing their hold in these areas by constructing their own houses
Banerjee, 2019 [112]				Dwellers are mostly from lower social and economic status and remain more exposed to the physically demanding jobs, poorer health status, and associated disabilities
Adams, 2020 [107]		limited access to basic services	Inadequate housing	Crowding, insecurity

### Economic burden

Most studies estimated direct costs (medical and/or non-medical) of accessing healthcare and only 19 (30%) estimated both direct and indirect costs (Table 2). Cost outcomes were provided in different ways (mean, median and cost per capita; cost of an illness episode; cost of outpatient or inpatient care; cost of the last visit to a health provider) and time horizons (fortnight, month, year). Table S5 reports costs for all studies individually. The incidence of CHE was reported by 37 (58%) studies using different thresholds (ranging from 5 to 40%) and methods in estimation, such as household consumption [113, 114], household income [115–117], household capacity to pay [118–120], household non-food expenditure [36, 121] and total household expenditure [122] (Table S6 in supporting information). The benefit of enrolling in health insurance schemes was included in the cost analysis of 21 (33%) studies using the following approaches: costs adjusted by health insurance coverage, costs reported by health insurance coverage, the potential benefit of health insurance to protect from high costs or CHE, and premium for health insurance as a cost component.

### Cost of acute conditions

We found four comparable observations to estimate the direct costs of acute conditions. Slum studies reported higher median direct costs, particularly for the wealthier quintiles when compared with city-wide studies. The median direct cost of an episode of an acute condition ranged from I$157 (IQR: 79–236; poorest quintile) to I$408 (IQR: 67–749; richest quintile) in slum studies and from I$125 (IQR: 28–221; poor quintile) to I$177 (IQR: 41–313; richest quintile) in city-wide studies (Table 3).

**Table 3:** Mean (SD) and median (IQR) direct costs health interest, area of residence and wealth quintile. International Dollar, 2020 prices

Health interest	Q1 (Poorest)		Q2 (poor)		Q3 (middle)		Q4 (richer)		Q5 (Richest)	
	**Median (IQR)**	**Mean (SD)**	**Median (IQR)**	**Mean (SD)**	**Median (IQR)**	**Mean (SD)**	**Median (IQR)**	**Mean (SD)**	**Median (IQR)**	**Mean (SD)**
***Acute conditions***^***1***^										
* Slum studies (N* = *2)*	**157** **(79–236)**	157 (111)	**180** **(83–276)**	180 (136)	**182** **(87–276)**	182 (134)	**248** **(102–394)**	248 (206)	**408** **(67–749)**	408 (482)
* City-wide studies (N* = *2)*	**148** **(33–263)**	148 (163)	**125** **(28–221)**	125 (136)	**136** **(29–242)**	136 (151)	**133** **(34–232)**	133 (140)	**177** **(41–313)**	177 (192)
***Chronic conditions***^***2***^										
* Slum studies (N* = *3)*	**789** **(442–930)**	720 (251)	**845** **(555–1,313)**	904 (382)	**906** **(645–916)**	822 (135)	**1,001** **(865–1,234)**	1,033 (186)	**1,695** **(721–1,994)**	1,470 (666)
* City-wide studies (N* = *4)*	**2,870** **(828–5,653)**	3,241 (3,070)	**2,552** **(940–5,342)**	3,141 (3,042)	**3,166** **(1,242–5,848)**	3,545 (3,131)	**2,921** **(1,138–5,807)**	3,472 (3,223)	**2,607** **(959–6,354)**	3,656 (3,830)
***Unspecified health conditions***^***2***^										
* Slum studies (N* = *3)*	**451** **(91–663)**	402 (289)	**440** **(78–515)**	344 (233)	**365** **(96–666)**	376 (285)	**378** **(88–527)**	331 (223)	**343** **(105–576)**	341 (235)
* City-wide studies (N* = *21)*	**307** **(134–551)**	561 (869)	**502** **(158–682)**	607 (669)	**458** **(207–1,030)**	704 (830)	**577** **(338–1,433)**	1,529 (2,340)	**967** **(211–2,322)**	1,421 (1,373)

*N* number of observations

^1^Cost of the last disease episode or care

^2^Cost incurred during one-year time horizon

### Cost of chronic conditions

We found seven comparable observations to estimate the direct costs of chronic conditions. Compared with slum studies, city-wide studies reported higher median direct costs for the treatment of chronic conditions in all wealth quintiles. Costs of one-year treatment for chronic conditions ranged from I$789 (IQR: 442–930; poorest quintile) to I$1,695 (IQR: 721–1,994, richest quintile) for slum studies, and from I$2,552 (IQR: 940–5,342; poor quintile) to $3,166 (IQR: 1,242–5,848; middle quintile) for city-wide studies (Table 3).

### Cost of unspecified health conditions

We found 24 comparable observations to estimate direct costs of unspecified health conditions. Median direct costs of one-year treatment for unspecified health conditions ranged from I$343 (IQR: 105–576; richest quintile) to I$451 (IQR: 91–663, poorest quintile) for slum studies, and from I$307 (IQR: 134–551; poorest quintile) to I$967 (IQR: 211–2,322; richest quintile) for city-wide studies. Except for the poorest wealth quintile, city-wide studies reported higher median costs than slum studies (Table 3).

### Progressiveness and equity of healthcare expenditures

**Fig. 2:**
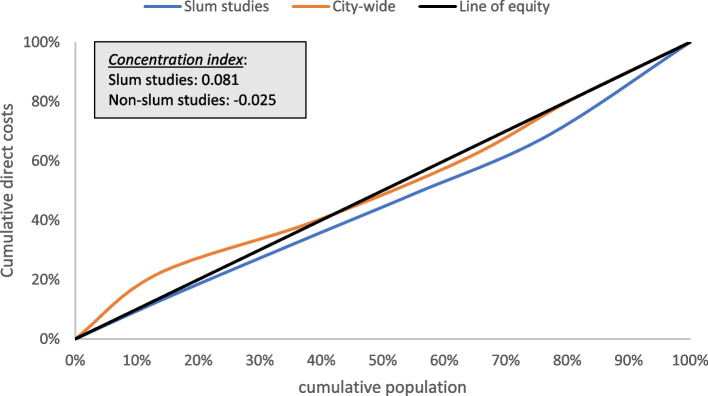
Concentration curves and indexes for health-care expenditure in slums and city-wide studies for chronic conditions, one-year time horizon.

We found five observations providing costs and sample size per wealth quintile to compute concentration curves and indices for direct costs of chronic conditions. The concentration curves indicated that the cost of accessing healthcare for chronic conditions was concentrated among the richest wealth quintiles for the slum studies as the concentration curves are placed below the line of equality. For city-wide studies, the concentration curve had a mixed pattern with direct costs more equally distributed across all wealth quintiles. The concentration index reflecting socioeconomic-related inequities in healthcare expenditure indicated a progressive pattern for slum studies (positive concentration index, 0.081), that is expenditures rise as a proportion of income rises. In city-wide studies, the concentration index (negative concentration index, -0.025) indicated a weakly regressive pattern (i.e. expenditures concentrated amongst the poorest) as the cost of the poorest and richest wealth quintiles are similar (Fig. 2).

#### Catastrophic health expenditures

**Fig. 3:**
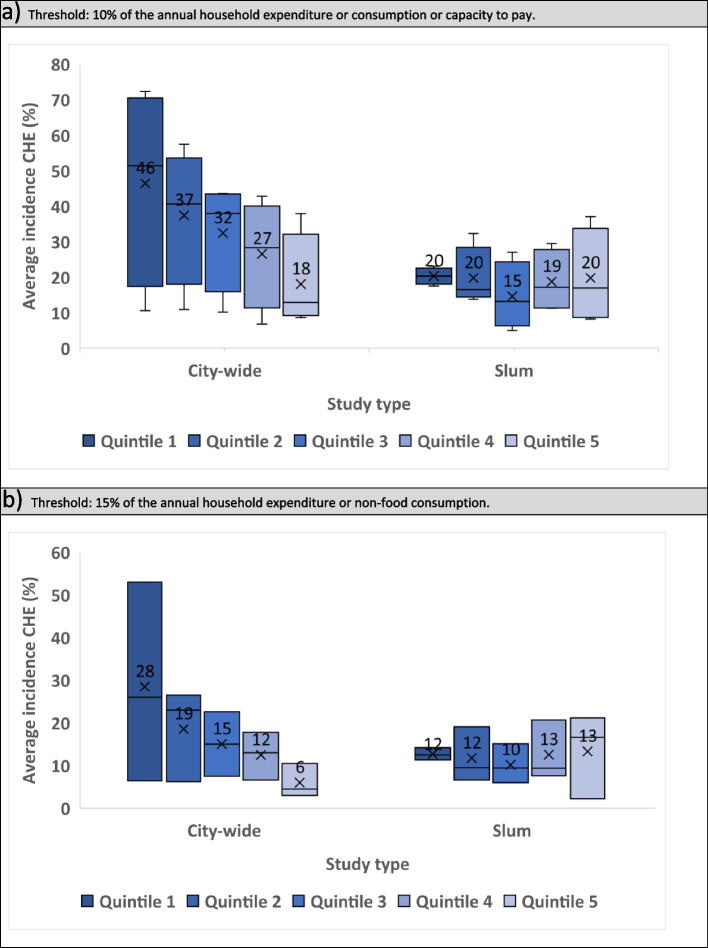
Average incidence of catastrophic health expenditures for chronic conditions and general health care by study type and wealth quintile (1=poorest; 5=richest).

We found eight observations providing CHE per wealth quintile using a 10% threshold and six using a 15% threshold. The mean incidence of CHE in accessing healthcare for chronic and acute conditions using a 10% threshold was similar across all wealth quintiles for slum studies (Q1, Q2, Q5: 20%; Q3: 15%; Q4: 19%). For city-wide studies, the poorest incurred more CHE, the average incidence of CHE in Q1 was more than double that in Q5 (46% vs 18%). City-wide studies also presented a higher incidence of CHE when compared with slum studies in all wealth quintiles. We observed similar patterns using the 15% threshold (Fig. 3).

#### Evidence map and quality assessment

**Fig. 4:**
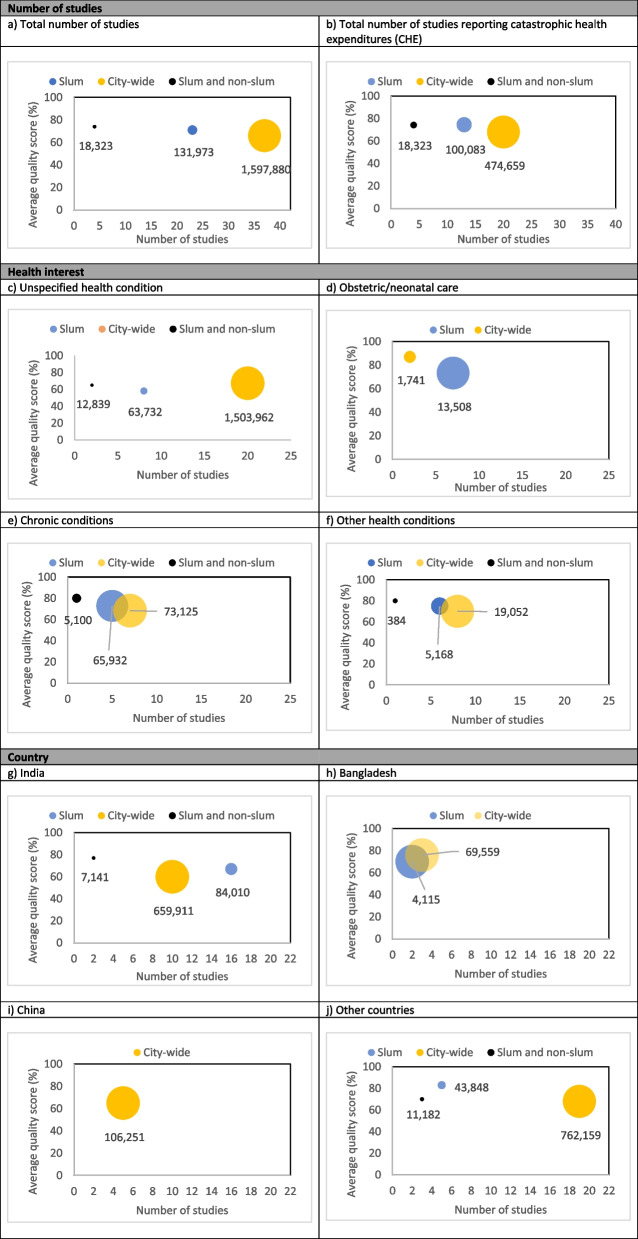
Evidence map and strength of evidence of studies. The size of the bubbles indicates the total sample size (number of individuals), the X axis indicates the total number of studies and the Y axis indicate the strength of evidence (good: >85%, moderate: 75% - 84%, poor: <75%).

City-wide studies reached a higher number of publications and a larger total sample size (more than 1.5 million individuals) when compared with slum and slum/non-slum studies (Figs. 4a). City-wide studies had more publications with CHE calculated, with 20 publications compared with 13 for slum and 4 from slum/non-slum studies (Fig. 4b). City-wide studies dominated evidence on unspecified and other health conditions (i.e. injuries, acute disease, child health and tuberculosis), while slum studies dominated the evidence on obstetric and/or neonatal care. For chronic conditions, city-wide studies had more publications, but the total sample size was similar for city-wide and slum studies (~ 70,000 individuals) (Fig. 4c-4f). Analysing the evidence by country, we found dominance of city-wide studies in the number of publications and/or total sample size in all countries (Figs. 4 g-4j).

The quality of evidence was mostly classified as poor. Slum studies reached moderate quality on the topics CHE (75%), other health conditions (75%) and other countries (83%) (Figs. 4b, 4f and 4j). City-wide studies had good quality on the topics of obstetric and neonatal care (87%) and moderate quality in Bangladesh (76%) (Figs. 4d and 4 h). Slum/non-slum studies had moderate quality on the topics of chronic conditions (80%), other health conditions (80%) and India (77%) (Figs. 4e, 4f and 4 g).

In general, studies did not report the sample size justification, power description, variance and effect estimates; the method for adjusting unit costs to the reporting year and performing currency conversion; and the denominator or refusals, or incomplete forms. Half of the studies that included indirect costs in the estimates did not report the method used for valuing productivity loss (Table S7).

## Discussion

Our findings indicate a severe economic burden of accessing healthcare for both slum and city-wide residents, particularly amongst the poorest urban populations. However, we observed important differences in healthcare expenditures and economic burden when comparing health conditions, wealth quintiles and urban populations.

These different patterns of healthcare expenditures and economic burden may have several causes. An initial consideration is a relationship between income and health [37]. Evidence has shown that the rising income leads to the purchase of more and usually better-quality health services [123, 124]. Also, an analysis of healthcare use and expenditure across public and private providers from eight LMICs found that richer groups were more likely to access care when sick, to be seen by a doctor, and receive medicines when ill than the poorer groups [123]. This can explain the high costs amongst the better-off urban residents (5^th^ wealth quintile) and higher costs for city-wide (better-off) compared with slum (poorer) residents for some health conditions.

Analysis of healthcare services in slum areas suggests that poor people with chronic illnesses have more difficulties in accessing healthcare due to the lack of medical providers nearby [20, 125]. The opening time of health facilities has also been reported as a barrier to healthcare access in many cities in LMIC [19]. This explains the lower costs of chronic conditions for slum-dwellers compared with city-wide residents.

Another important element that influences healthcare expenditures is the growing trend in the use of private services through non-governmental organizations and informal providers in slum settlements [17, 126, 127], which may lead to the high cost of care for acute conditions for slum-dwellers. This trend was observed for obstetric care in Nairobi, Kenya where women residents of poor settlements were more likely to give birth in private services compared with women residents of better-off settlements [127].

The poor access to the formal health sector, multiple health providers used by slum residents over long periods, and choices and preferences for healthcare providers also affect healthcare expenditures [17, 128, 129]. A study conducted in Brazilian slums found that the formal sector reaches the vulnerable slum residents with a deteriorated health status due to the lack of adequate assistance during the initial stages of illness. The poor quality and access to healthcare also led to higher costs for these populations compared with non-slum residents [128].

Our findings indicated different distribution and proportions of households affected by CHE across wealth quintiles and study settings. A multi-country analysis of CHE specified three main factors influencing CHE, the availability of health services requiring payment, low capacity to pay, and the lack of prepayment or health insurance [119]. We did not analyse the influence of these specific factors in our results as this is out of the scope of this review. But they may explain the higher incidence of CHE amongst the better-off urban residents. Seeberg et al. (2014) also discussed and compared CHE incurred by slum and city-wide dwellers in India, Thailand and Indonesia. The authors highlighted that the poorest patients in slums may not be able to generate enough funding for the treatment of their health conditions and so are less likely to incur CHE [106].

In line with other commentators [3, 4], we found great variation in the definitions used to characterize slum settlements in our included studies. These differences impact our understanding of the determinants of a range of health outcomes as illustrated by a comparative analysis of the impact of slum definitions on the identification of the determinants and extent of insufficient child height and weight for age in Indian slums [4]. A recent scoping review suggested a combination of household and area-level data to measure deprivation and to be used for comparison between different cities in LMICs. The review showed that deprivation frameworks can help to identify the degree of poverty in a community and guide health policies in slum areas [3]. Future debates on slum definition considering the complexity of these entities must be included in the urban health agenda to inform public policies.

The evidence map uncovered several gaps in the research addressing the economic burden of healthcare access on the urban poor. Almost half of the studies took place in India, and more than half were classified as city-wide studies. Our search did not identify any slum or city-wide studies from countries with a high density of urban slums, such as the Central African Republic (95%), South Sudan (91%), Sudan (88%), Chad (87%) and Sao Tome and Principe (86%) [8]. Overall, the average strength of evidence was poor and lacked information on key methodological aspects. City-wide studies seem to be more complete in terms of cost analysis as they reported economic burden in terms of costs and CHE more frequently than slum studies. Obstetric and neonatal care was the only health interest that we found more slum than city-wide studies.

This review has some limitations with some of them intrinsic to the stages of development of scoping reviews as indicated by Levac et al. (2010) [24]. Our broad research questions and inclusion of multiple languages led to a high number of studies identified by the search strategy. The feasibility of screening such a high number of studies whilst also building the capacity of team members new to systematic reviews led to several discordances in the first and second reviewer screening. These discordances were extensively discussed during the review meetings and agreement was reached by consensus. A high number of discordances were also reported in the data extraction process and some studies were excluded at this stage because they did not meet the inclusion criteria. While this ultimately delayed the next stages of the review it did provide a valuable mechanism for applied capacity strengthening on systematic review methods.

Other limitations related to the data analysis were identified. First, our findings indicated the economic burden of direct costs only (direct medical and non-medical expenditures). The analysis of indirect costs, which was not included in this review due to the lack of comparable costing outcomes, might show different patterns of economic burden. For example, income loss usually affects the most vulnerable populations in developing countries, that is those employed in the informal market with no protection from labour legislation [130]. Therefore, the inclusion of indirect costs would probably increase the economic burden of healthcare for the poorest urban residents. Second, some analyses proposed in the original protocol could not be performed due to the reduced number of comparable outcomes. Third, concentration curves and indices were computed only for chronic health conditions also due to the small number of comparable results. Fourth, our estimates must be interpreted with caution as the included studies adopted different methods to calculate costs and CHE, which can lead to less robust estimates. We also combined chronic and unspecified health conditions to show trends in the pattern of CHE. Fifth, our findings are skewed geographically with most cost estimates coming from countries in the South-East Asian Region, particularly from India, our results are likely to reflect the scenario in that region. Lastly, the economic burden reported in this review may be underestimated as those living in severe deprivation may not search for healthcare due to the lack of financial resources. These patients may report ‘zero direct costs’ of accessing healthcare, but they still suffer the economic consequences of untreated diseases such as loss of productivity and deteriorated quality of life.

Our review also has strengths. Our team included researchers from different backgrounds and skills, including health economists, statisticians, epidemiologists, urban health specialists and social scientists. We adopted a collaborative approach which allowed a cross-learning process among different disciplines. All reviewers from the core and new reviewer teams completed the Cochrane interactive learning on systematic reviews [131] to ensure high quality during all steps of the review. We also set up regular meetings to discuss each step of the review, methods applied and the use of web tools such as Rayyan and COVIDENCE. Additionally, we reviewed articles written in English, Chinese, Spanish, French and Portuguese to cover health systems in different countries.

## Conclusion

Our review revealed severe economic burden and different patterns of healthcare expenditures when comparing wealth quintiles and urban populations in LMICs. The findings indicate that the urban poor, both in slums and in poor households dispersed across cities, need to be protected from the severe effects of high costs and CHE through financial and risk protection policies. These policies must consider the complexity and variety of healthcare provision in the urban context. As an exploratory study, this scooping review identified important gaps in terms of methodological quality of the studies, heterogeneity of cost analysis, and research development in slum environments that must be addressed in future studies.

## Supplementary Information

**Additional file 1:Table S1.** Inclusion and exclusion criteria of the scoping review. **FigureS1.** Flow chart describing inclusion and exclusion pathways. **Table S2.** DataExtraction form. **Table S3.** Qualityappraisal tool. **Table S4.** Description of health conditions. **Table S5.** Critical findings and individualcosts. NR= Not reported; CHE= catastrophic health expenditures; Q1 to Q5=wealth quintile (from poorest to richest); Q1 to Q4= wealth quartile (frompoorest to richest); T1 to T3= wealth tertial (from poorest to richest); D1 to D2=wealth decile (poor, non-poor or below, above poverty line). **Table S6.** Characteristics of theCost-analysis CHE= Catastrophic health expenditure. **Table S7.** Quality assessment

## Data Availability

All data generated or analysed during this study are included in this published article [and its supplementary information files].

## Abbreviations

CHE: Catastrophic health expenditure
LMICs: Low- middle-income countries
NGO: Non-government organisation
CHEERS: Consolidated Health Economic Evaluation Reporting Standards
NIH: National Heart, Lung and Blood Institute
SD: Standard deviation
IQR: Interquartile range
