# Supplementary material for: The economics of healthcare access: a scoping review on the economic impact of healthcare access for vulnerable urban populations in low- and middle-income countries
Source: Int J Equity Health. 2022 Dec 31;21:191. doi: 10.1186/s12939-022-01804-3 (PMC9805259; doi:10.1186/s12939-022-01804-3)
Supplement: Supplementary file 1 — Additional file 1:Table S1. Inclusion and exclusion criteria of the scoping review. FigureS1. Flow chart describing inclusion and exclusion pathways. Table S2. DataExtraction form. Table S3. Qualityappraisal tool. Table S4. Description of health conditions. Table S5. Critical findings and individualcosts. NR= Not reported; CHE= catastrophic health expenditures; Q1 to Q5=wealth quintile (from poorest to richest); Q1 to Q4= wealth quartile (frompoorest to richest); T1 to T3= wealth tertial (from poorest to richest); D1 to D2=wealth decile (poor, non-poor or below, above poverty line). Table S6. Characteristics of theCost-analysis CHE= Catastrophic health expenditure. Table S7. Quality assessment [file 12939_2022_1804_MOESM1_ESM.docx]

**Supplementary material**

*Search strategy*

The search strategies for MEDLINE incorporated a low- and middle-income countries (LMIC) search filter developed by the Effective Practice and Organisation of Care (EPOC) Cochrane group.^1^ Where possible, this filter was translated for use in the other databases searched.

Search date: 22^nd^ June 2020

Records retrieved: 2368

POPULATION

1 Urban Population/ (58856)

2 Cities/ (19180)

3 Urban Health/ (17793)

4 Urban Health Services/ (3613)

5 Hospitals, Urban/ (7341)

6 urban$.ti,ab. (156001)

7 (city or cities or megacity or megacities or metropolitan).ti,ab. (169349)

8 (metropolis or megalopolis or municipal$ or conurbation$ or suburb$ or town or towns or township$ or borough$ or barrio or barrios).ti,ab. (81323)

9 ((dense$ or density or high$ or large$ or heavy or heavily) adj3 (populat$ or populous) adj3 (area$ or settlement$ or district$ or neighbourhood$ or communit$)).ti,ab. (3451)

10 or/1-9 (390327)

11 (afghanistan or albania or algeria or american samoa or angola or "antigua and barbuda" or antigua or barbuda or argentina or armenia or armenian or aruba or azerbaijan or bahrain or bangladesh or barbados or republic of belarus or belarus or byelarus or belorussia or byelorussian or belize or british honduras or benin or dahomey or bhutan or bolivia or "bosnia and herzegovina" or bosnia or herzegovina or botswana or bechuanaland or brazil or brasil or bulgaria or burkina faso or burkina fasso or upper volta or burundi or urundi or cabo verde or cape verde or cambodia or kampuchea or khmer republic or cameroon or cameron or cameroun or central african republic or ubangi shari or chad or chile or china or colombia or comoros or comoro islands or iles comores or mayotte or democratic republic of the congo or democratic republic congo or congo or zaire or costa rica or "cote d’ivoire" or "cote d’ ivoire" or cote divoire or cote d ivoire or ivory coast or croatia or cuba or cyprus or czech republic or czechoslovakia or djibouti or french somaliland or dominica or dominican republic or ecuador or egypt or united arab republic or el salvador or equatorial guinea or spanish guinea or eritrea or estonia or eswatini or swaziland or ethiopia or fiji or gabon or gabonese republic or gambia or "georgia (republic)" or georgian or ghana or gold coast or gibraltar or greece or grenada or guam or guatemala or guinea or guinea bissau or guyana or british guiana or haiti or hispaniola or honduras or hungary or india or indonesia or timor or iran or iraq or isle of man or jamaica or jordan or kazakhstan or kazakh or kenya or "democratic people’s republic of korea" or republic of korea or north korea or south korea or korea or kosovo or kyrgyzstan or kirghizia or kirgizstan or kyrgyz republic or kirghiz or laos or lao pdr or "lao people's democratic republic" or latvia or lebanon or lebanese republic or lesotho or basutoland or liberia or libya or libyan arab jamahiriya or lithuania or macau or macao or "macedonia (republic)" or macedonia or madagascar or malagasy republic or malawi or nyasaland or malaysia or malay federation or malaya federation or maldives or indian ocean islands or indian ocean or mali or malta or micronesia or federated states of micronesia or kiribati or marshall islands or nauru or northern mariana islands or palau or tuvalu or mauritania or mauritius or mexico or moldova or moldovian or mongolia or montenegro or morocco or ifni or mozambique or portuguese east africa or myanmar or burma or namibia or nepal or netherlands antilles or nicaragua or niger or nigeria or oman or muscat or pakistan or panama or papua new guinea or new guinea or paraguay or peru or philippines or philipines or phillipines or phillippines or poland or "polish people's republic" or portugal or portuguese republic or puerto rico or romania or russia or russian federation or ussr or soviet union or union of soviet socialist republics or rwanda or ruanda or samoa or pacific islands or polynesia or samoan islands or navigator island or navigator islands or "sao tome and principe" or saudi arabia or senegal or serbia or seychelles or sierra leone or slovakia or slovak republic or slovenia or melanesia or solomon island or solomon islands or norfolk island or norfolk islands or somalia or south africa or south sudan or sri lanka or ceylon or "saint kitts and nevis" or "st. kitts and nevis" or saint lucia or "st. lucia" or "saint vincent and the grenadines" or saint vincent or "st. vincent" or grenadines or sudan or suriname or surinam or dutch guiana or netherlands guiana or syria or syrian arab republic or tajikistan or tadjikistan or tadzhikistan or tadzhik or tanzania or tanganyika or thailand or siam or timor leste or east timor or togo or togolese republic or tonga or "trinidad and tobago" or trinidad or tobago or tunisia or turkey or "turkey (republic)" or turkmenistan or turkmen or uganda or ukraine or uruguay or uzbekistan or uzbek or vanuatu or new hebrides or venezuela or vietnam or viet nam or middle east or west bank or gaza or palestine or yemen or yugoslavia or zambia or zimbabwe or northern rhodesia or global south or africa south of the sahara or sub-saharan africa or subsaharan africa or africa, central or central africa or africa, northern or north africa or northern africa or magreb or maghrib or sahara or africa, southern or southern africa or africa, eastern or east africa or eastern africa or africa, western or west africa or western africa or west indies or indian ocean islands or caribbean or central america or latin america or "south and central america" or south america or asia, central or central asia or asia, northern or north asia or northern asia or asia, southeastern or southeastern asia or south eastern asia or southeast asia or south east asia or asia, western or western asia or europe, eastern or east europe or eastern europe or developing country or developing countries or developing nation? or developing population? or developing world or less developed countr* or less developed nation? or less developed population? or less developed world or lesser developed countr* or lesser developed nation? or lesser developed population? or lesser developed world or under developed countr* or under developed nation? or under developed population? or under developed world or underdeveloped countr* or underdeveloped nation? or underdeveloped population? or underdeveloped world or middle income countr* or middle income nation? or middle income population? or low income countr* or low income nation? or low income population? or lower income countr* or lower income nation? or lower income population? or underserved countr* or underserved nation? or underserved population? or underserved world or under served countr* or under served nation? or under served population? or under served world or deprived countr* or deprived nation? or deprived population? or deprived world or poor countr* or poor nation? or poor population? or poor world or poorer countr* or poorer nation? or poorer population? or poorer world or developing economy* or developing economies or less developed econom* or lesser developed econom* or under developed econom* or underdeveloped econom* or middle income econom* or low income econom* or lower income econom* or low gdp or low gnp or low gross domestic or low gross national or lower gdp or lower gnp or lower gross domestic or lower gross national or lmic or lmics or third world or lami countr* or transitional countr* or emerging economy or emerging economies or emerging nation?).ti,ab,sh,kf. (1907989)

12 (afghan or afghans or afghani or albanian? algerian? or american samoan? or angolan? or antiguan? or barbudan? or argentine? or argentinian? or argentinean? or armenian? or aruban? or azerbaijani? or bahraini? or bangladeshi? or bangalees or bajan? or belarusian? or byelorussian? or belizean? or beninese? or bhutanese or bolivian? or bosnian? or botswana or batswana or brazilian? or brasilian? or bulgarian? or burkinabe or burkinese or burundian? or cape verdean? or cabo verdean? or cambodian? or khmer or cameroonian? or central african? or chadian? or chilean? or chinese or colombian? or comorian? or congolese or costa rican? or ivorian? or croatian? or cuban? or cypriot? or czech? or djiboutian? or dominican? or ecuadorian? or egyptian? or salvadoran? or equatorial guinean? or equatoguinean? or eritrean? or estonian? or swazi? or swati? or ethiopian? or fijian or gabonese or gabonaise or gambian? or georgian? or ghanaian? or gibraltarian? or greek? or grenadian? or guamanian? or guatemalan? or guinean? or bissau guinean? or guyanese or haitian? or honduran? or hungarian? or indian? or indonesian? or iranian? or iraqian? or iraqi? or manx or jamaican? or jordanian? or kazakhstani? or kenyan? or kirabati or kirabatian? or north korean? or korean? or kosovar? or kosovan? or kyrgyz* or lao or laotian? or latvian? or lebanese or lesothan? or lesothonian? or mosotho or basotho or liberian? or libyan? or lithuanian? or macanese or macedonian? or malagasy or madagascan? or malawian? or malaysian? or maldivian? or malian? or maltese or marshallese? or mauritanian? or mauritian? or mexican? or micronesian? or moldovan? or mongolian? or mongol or montenegrin? or moroccan? or mozambican? or burmese or myanma or namibian? or nauruan? or nepali or nepalese or netherlands antillean? or nicaraguan? or nigerien? or nigerian? or northern mariana islander? or mariana? or omani? or pakistani? or palauan? or panamanian? or papua new guinean? or paraguayan? or peruvian? or philippine? or philipine? or phillipine? or phillippine? or filipino? or filipina? or polish or pole or poles or portuguese or puerto rican? or romanian? or russian? or soviet people or soviet population or rwandan? or rwandese or ruandan? or ruandese or samoan? or sao tomean? or santomean? or saudi arabian? or saudi? or senegalese or serbian? or montenegrin? or seychellois or seychelloise? or sierra leonean? or slovak? or slovene? or solomon islander? or somali? or south african? or south sudanese or sri lankan? or ceylonese or kittitian? or nevisian? or saint lucian? or vincentian? or sudanese or surinamese? or syrian? or tajik? or tajikistani? or tanzanian? or tanganyikan? or thai or timorese? or togolese or tongan? or trinidadian? or tobagonian? or tunisian? or turk? or turkish or turkmen? or tuvaluan? or ugandan? or ukrainian? or uruguayan? or uzbek? or vanuatu* or venezuelan? or vietnamese or yemeni? or yemenite? or yemenese or yugoslav? or yugoslavian? or zambian? or zimbabwean?).ti,ab,sh,kf. (834927)

13 11 or 12 (2269396)

14 10 and 13 (169934)

COSTS

15 poverty areas/ (6047)

16 (slum or slums or ghetto or ghettos or shanty$ or shanties or shack$ or favela$).ti,ab. (5200)

17 (informal$ adj3 settlement$).ti,ab. (558)

18 ((poverty or impoverish$) adj3 (area$ or settlement$)).ti,ab. (881)

19 ((precarious$ or irregular$) adj3 settlement$).ti,ab. (8)

20 (squatter$ adj3 (area$ or settlement$)).ti,ab. (227)

21 or/15-20 (11156)

22 "cost of illness"/ (27054)

23 exp Health Care Costs/ (64997)

24 Health Expenditures/ (20173)

25 exp Financing, Personal/ (6053)

26 Catastrophic Illness/ec [Economics] (550)

27 (cost$ adj2 (illness$ or sickness$ or disease$)).ti,ab. (5084)

28 (cost$ adj3 (care or health or healthcare or medical$)).ti,ab. (76646)

29 (expenditure$ adj3 (care or health or healthcare or medical$)).ti,ab. (13957)

30 ((direct or indirect) adj2 (cost$ or expenditure$)).ti,ab. (15577)

31 out of pocket.ti,ab. (5698)

32 (OOP adj3 (cost$ or expense$ or expenditure$ or financ$ or pay$ or paid or spend$)).ti,ab. (505)

33 OOPE.ti,ab. (74)

34 ((personal or individual$ or patient$ or family$ or families or household$) adj3 (budget$ or cost$ or expense$ or expenditure$ or financ$ or pay$ or paid or spend$) adj3 (care or health or healthcare or medical$)).ti,ab. (9512)

35 (catastroph$ adj10 (cost$ or expense$ or expenditure$ or financ$ or pay$ or paid or spend$)).ti,ab. (1323)

36 ((medical$ or health) adj3 impoverish$).ti,ab. (232)

37 (burden$ adj3 (cost$ or expenditure$ or economic$ or financ$) adj3 (care or health or healthcare or medical$)).ti,ab. (4485)

38 ((willing$ or unwilling$) adj3 pay$ adj3 (care or health or healthcare or medical$)).ti,ab. (323)

39 ((afford$ or unafford$) adj5 (cost$ or pay$)).ti,ab. (2273)

40 ((able or ability or capacit$ or unable or inability) adj2 pay$).ti,ab. (1460)

41 or/22-40 (185296)

42 14 and 41 (3361)

43 21 and 41 (267)

44 42 or 43 (3541)

TYPE OF STUDY

45 letter.pt. (1084673)

46 editorial.pt. (532516)

47 historical article.pt. (358723)

48 or/45-47 (1956361)

49 44 not 48 (3506)

50 exp animals/ not humans/ (4709143)

51 49 not 50 (3497)

52 limit 51 to yr="2010 -Current" (2368)

**Key:**

/ = subject heading (MeSH heading)

exp = exploded subject heading (MeSH heading)

/ec = economics subheading

$ = truncation

* = truncation

“ “ = exact phrase search

? = stands for zero or one characters within a word or at the end of a word

ti,ab = terms in either title or abstract fields

kf = terms in author keywords field

sh = terms in the subject heading field

pt = terms in publication type field

adj3 = terms within three words of each other (any order)

adj = terms next to each other in the same order

Table S1. Inclusion and exclusion criteria of the scoping review.

| **Appraisal** | **Inclusion Criteria** | **Exclusion Criteria** |
| --- | --- | --- |
| Country | Low and middle-income countries | High income countries |
| Article type | Peer review articles, theses, dissertations, working papers and reports | Case reports, protocols, news article, editorial, conference abstracts, comments |
| Study design | Cost and cost of illness studies | Willingness to pay, health finance and economic evaluations addressing the provider perspective, modelling studies using secondary cost data, systematic and scoping reviews |
| Focus of study | Access to health care: NGOs, private and public sector, outpatient and inpatient care. Studies that disaggregate by slum/non-slum or wealth quintile/poor/non-poor; studies that are slum specific | Studies that do not disaggregate between rural and urban or urban wealth categories; studies focused on rural areas |

Figure S1. Flow chart describing inclusion and exclusion pathways

Table S2. Data Extraction form

| **General information** | - Lead author and year - Country in which the study conducted - Geographical area where the study took place - Classification of the study setting in slum or non-slum area - Definition of slum given by the study |
| --- | --- |
| **Methodological characteristics** | - Study design - Study population - Health interest - Population covered by health insurance - Cost analysis considered health insurance coverage - Type of health insurance - Sampling method - Total number of participants - Sample size by the main comparison group - Sample size by wealth categories (quintile, quartile and others) - Method applied to calculate wealth categories - Primary data source - Period of data collection - Type of health care provider - Level of health providers - Tool(s) to collect data/sources of data - Unit of analysis Measure of spending/payment - Type of costs included - Cost analysis - Currency - Conversion rate - Year of conversion rate - Method to calculate catastrophic costs - Threshold(s) of catastrophic costs - Cost disaggregated by wealth quintile - Wealth disaggregation |
| **Results** | - Disaggregated/ aggregated catastrophic cost - Level of disaggregation - Disaggregated/ aggregated direct medical costs - Disaggregated/ aggregated direct non-medical costs - Disaggregated/ aggregated indirect costs - Disaggregated/ aggregated total costs |

Table S3. Quality appraisal tool

| **Instrument** | **Questions** |
| --- | --- |
| NIH | 1. Was the research question or objective in this paper clearly stated? 2. Was the sampling method described and adequate? 3. Was a sample size justification, power description, or variance and effect estimates provided? 4. Were inclusion and exclusion criteria described and adequate? 5. Was the participation rate of eligible persons at least 50%? 6. Was the study population clearly described? 7. Was denominator or refusals, or incomplete forms adequately reported? |
| CHEERS | 1. Was the period of data collection reported? 2. Was the study setting clearly described? 3. Were types of costs, out of pocket expenditures clearly defined? 4. When included, was the method used for valuing productivity losses explained and justified? 5. Was the method for adjusting unit costs to the reporting year and performing currency conversion explained? 6. Was the result analysed and reported for all included participants? |
| TBCA | 1. Is there a clear description of patient interview procedures given? |
| New questions | 1. Was the methodology applied to calculate income level described and adequate? 2. When included, was the method used for estimating catastrophic costs explained? |

Table S4. Description of health conditions

| Acute conditions | Child care, diseases or symptoms such as skin diseases, measles, respiratory infections, malaria, common cold and gastro-intestinal disorders diarrhoea, road traffic injuries, delivery, post-partum and neonatal care, dental care |
| --- | --- |
| Chronic conditions | Cardiovascular, digestive, endocrine, musculoskeletal, neurological and respiratory diseases, diabetes, cancer, hypertension, AIDS |
| Unspecified health conditions | Inpatient and/or outpatient care with no information of the health condition |

Table S5. Critical findings and individual costs.

| **Reference** | **Data source, tool** | **Type and level of provider** | **Costs (International Dollar, 2020 prices)** |
| --- | --- | --- | --- |
| Das, 2010 | - Interviews - Predominantly closed questionnaire | - Public, Private, Informal (traditional healer) - Primary, secondary and tertiary | Median direct cost of obstetric/neonatal care in Mumbai, public sector: 119  Median direct cost of obstetric/neonatal care in Mumbai, private sector: 596  Median direct cost of obstetric/neonatal care outside Mumbai, public sector: 119  Median direct cost of obstetric/neonatal care outside Mumbai, private sector: 331  Median indirect cost of obstetric/neonatal care in Mumbai, public sector: 60  Median indirect cost of obstetric/neonatal care in Mumbai, private sector: 60  Median indirect cost of obstetric/neonatal care outside Mumbai, public sector: 48  Median indirect cost of obstetric/neonatal care outside Mumbai, private sector: 51 |
| Barros, 2011 | - National survey, interviews - 2002-2003 Brazilian Household Budget Survey | NR | Only CHE reported |
| Lopera, 2011 | - Interviews - Structured questionnaire guided by a literature review, key informants, and input from experts | - NGO and public - Secondary, tertiary, emergency care | Direct costs  Mean cost of chronic conditions last year  Q1: 17; Q2: 29; Q3: 29; Q4: 41 |
| Garcia, 2012 | - National survey, interviews - Pesquisa de Orcamentos Familiares | - Public and private - Secondary and tertiary | Direct costs  Mean cost health care last year  Q1: 502; Q2: 502; Q3: 1,282; Q4: 2,204; Q5: 4,636 |
| Saini, 2012 | - Interviews | - Public, private, and Pharmacy, home delivery | Direct costs  Mean cost of antenatal care: 26  Mean cost of delivery in public sector: 206  Mean cost of delivery in private sector: 264  Mean cost of home delivery: 192 |
| Skordis-Worrall, 2012 | - Interviews | - Private and public maternity care | Direct costs  Mean cost maternal and neonatal care public sector  Q1: 79; Q2: 83; Q3: 87; Q4: 102; Q5: 67  Mean cost maternal and neonatal care private sector  Q1: 236; Q2: 276; Q3: 276; Q4: 394; Q5: 749  Indirect costs  Mean cost maternal and neonatal care public sector  Q1: 43; Q2: 32; Q3: 43; Q4: 32; Q5: 24  Mean cost maternal and neonatal care private sector  Q1: 39; Q2: 20; Q3: 20; Q4: 20; Q5: 39 |
| Bhojani, 2012 | - Interviews - Structured household-level questionnaire | - Public and private - Primary, secondary and tertiary | Only CHE reported |
| Kumar, 2012 | - Interviews - Structured questionnaire | - Public and private - Tertiary | Direct costs  Mean cos of injury, patient alive  Q1: 4,160; Q2: 3,904; Q3: 3,525; Q4: 6,860  Mean cos of injury, patient dead  Q1: 5,733; Q2: 5,305; Q3: 5,912; Q4: 8,308 |
| Weraphong, 2013 | - Interviews - Structured questionnaire adapted from the household survey questionnaires of the Centre for Health Equity Monitoring, Faculty of Medicine, Naresuan University | - Primary care units, private clinics, private hospitals, drug store, other government hospitals, regional hospitals - Primary, tertiary, drug stores | Mean cost of health care last year  Direct costs  D1: 485; D2: 474  Indirect costs  D1: 52; D2: 146 |
| Sarker, 2013 | - Interviews - Structured questionnaire | - Pharmacy, Private clinic, NGO, traditional practitioner, local formal physicians - Tertiary | Mean cost per episode of acute disease  Direct costs  Q1: 33; Q2: 28; Q3: 29; Q4: 34; Q5: 41  Indirect costs  Q1: 79; Q2: 149; Q3: 71; Q4: 114; Q5: 97  Total costs  Q1: 112; Q2: 177; Q3: 100; Q4: 148; Q5: 138 |
| Sakdapolrak, 2013 | - Interviews, symptoms diary - Symptoms diary, participatory action research | - Public, private, Informal (traditional healer) - Primary, secondary and tertiary | Mean cost of health care last year  Direct costs  Q1: 247; Q2: 157; Q3: 230; Q4: 242  Indirect costs  Q1: 147; Q2: 146; Q3: 183; Q4: 182  Total costs  Q1: 394; Q2: 303; Q3: 413; Q4: 424 |
| Misra, 2013 | - Interviews - Pre-structured, validated questionnaire in local language | - Public and private - Primary and secondary | Mean cost of health care access last 3 months  Total costs, baseline  Q1: 9; Q2: 18; Q3: 19; Q4: 42; Q5: 186  Total costs, follow-up  Q1: 107; Q2: 24; Q3: 34; Q4: 18; Q5: 48 |
| Rahman, 2013 | - Interviews - Bangladesh Household Income and expenditure Survey and Living Standards Measurement Survey | - Public, private, self-medication, traditional healer | Direct costs  Mean cost of chronic conditions last year  Q1: 1,265; Q2: 1,859; Q3: 2,467; Q4: 3,541; Q5: 5,629 |
| Patel, 2013 | - Interviews | - Private, pharmacy, public | Mean direct cost per episode of acute disease: 24  Mean indirect cost per episode of acute disease: 10 |
| Seeberg, 2013 | - Interviews - Modified version of an existing questionnaire | - Public, Private, Informal (Traditional healer), Pharmacy, NGO - Primary, secondary and tertiary | Only CHE reported |
| Ilesanmi, 2014 | - Interviews | - Secondary and tertiary | Only CHE reported |
| Wingfield, 2014 | - Interviews - Locally validated questionnaire | - Public - Primary | Mean cost of tuberculosis episode  Direct costs  T1: 252; T2: 320; T3: 333  Indirect costs: 435  Total costs: 735 |
| Chenge, 2014 | - Survey, interviews - Semi-structured interview | - Public, Private, Informal (Traditional healer), pharmacy - Primary, secondary and tertiary | Direct costs  Median costs of one illness episode last two weeks  Q1: 27; Q2: 29; Q3: 19; Q4: 25; Q5: 15 |
| Navneet, 2014 | - Interviews - A structured pre-tested schedule | - Public, private, Informal (traditional healer), charitable - Primary, secondary and tertiary | Direct costs  Median cost of health care last year: 275 |
| Saito, 2014 | - Interviews | - Public, private, traditional healers, pharmacy | Direct costs  Mean cost outpatient care last year  Q1: 1,321; Q2: 1,033; Q3: 1,030; Q4: 1,346; Q5: 1,798  Mean cost inpatient care last month  Q1: 1,387; Q2: 2,816; Q3: 1,435; Q4: 1,904; Q5: 2,755  Mean cost ayurvedic medicine last year  Q1: 199; Q2: 599; Q3: 547; Q4: 501; Q5: 838  Mean cost traditional medicine or healers last year  Q1: 174; Q2: 158; Q3: 229; Q4: 338; Q5: 211 |
| Rehman, 2014 | - Interviews | - Public, private, alternate medicine provider (pharmacy) | Direct costs  Mean cost of child care, last illness episode: 18 |
| Prabhakaran, 2014 | - Interviews - Pretested interview | - Mobile health clinic, private and other health facilities - Primary | Direct costs  Mean cost per visit for ante natal and pre-natal care: 9  Mean cost per visit to an outpatient mobile clinic: 3 |
| Tripathi, 2014 | - Interviews - Three part interview schedule developed for the study | - Public and private | Janani Shishu Suraksha Karyakram (JSSK)  Mean direct cost of antenatal care pre JSSK  D1: 428; D2: 366  Mean indirect cost of antenatal care pre JSSK: 110  Mean direct cost of antenatal care post JSSK  D1: 299; D2: 415  Mean indirect cost of antenatal care post JSSK: 102  Mean direct cost of delivery pre JSSK  D1: 460; D2: 421  Mean indirect cost of delivery pre JSSK: 86  Mean direct cost of delivery post JSSK  D1: 271; D2: 359  Mean direct cost of delivery post JSSK: 77 |
| Chandra, 2014 | - Interviews - Semi open-ended questionnaire | - Public and private | Direct costs  Mean cost of chronic conditions last year  Q1: 948; Q2: 528; Q3: 636; Q4: 1,200 |
| Joe, 2015 | - Survey, interviews | - Public and private - Primary, secondary and tertiary | Direct costs  Mean HH cost, hospitalisation last year  Q1: 641; Q2: 662; Q3: 767; Q4: 1,185; Q5: 2,014  Mean HH cots outpatient care last 15 days  Q1: 39; Q2: 38; Q3: 41; Q4: 49; Q5: 67 |
| da Silva, 2015 | - Interviews - Questionnaire | - Public and private - Primary, secondary and tertiary | Direct costs  Mean cost of child care last year  Q1: 59; Q2: 56; Q3: 60; Q4: 78; Q5: 106 |
| Patle, 2015 | - Interviews | - Traditional, government and non-government community health workers, allopathic practitioner | Direct costs  Mean cost of health care last year  D1: 63; D2: 153 |
| Putri, 2015 | - Interviews - Household survey | - Public hospital, private hospital, private doctor, public health centre, midwife - Secondary and tertiary | Direct costs  Mean cost of outpatient care last year  Q1: 42; Q2: 56; Q3: 63; Q4: 107; Q5: 68 |
| Thakare, 2015 | - Interviews - Survey questionnaire | - Public and private | Direct costs  Mean cost of chronic conditions public sector last year: 278  Mean cost of chronic conditions private sector last year: 524 |
| Buigut, 2015 | - Survey, interviews - Indicator Development for Surveillance of Urban Emergencies | - Public hospital, public clinic, private hospital, private clinic, mission hospital, mission clinic | Direct costs  Mean cost of health care last year  Q1: 148 |
| Davari, 2015 | - National survey, interviews - House-hold Income and Expenditure Survey in 2004 and 2011 | - Secondary and tertiary | Direct costs  Mean cost outpatient care last year, 2004  Q1: 551; Q2: 705; Q3: 1,090; Q4: 1,433; Q5: 0  Mean cost outpatient care last year, 2011  Q1: 214; Q2: 226; Q3: 180; Q4: 196  Mean cost inpatient care last year, 2004  Q1: 13; Q2: 682; Q3: 0; Q4: 125; Q5: 0  Mean cost inpatient care last year, 2011  Q1: 482; Q2: 427; Q3: 33; Q4: 6,761  Mean cost health care last year, 2004  Q1: 4,026; Q2: 3,022; Q3: 3,626; Q4: 2,873; Q5: 2,503  Mean cost health care last year, 2011  Q1: 1,222; Q2: 1,525; Q3: 1,846; Q4: 9,428 |
| Loganathan, 2015 | - Interviews and hospital bills - Standard, structured questionnaire developed for the study | - Public - Tertiary | Direct costs  Mean cost of child care, last illness episode:  Q1: 263; Q2: 221; Q3: 242; Q4: 232; Q5: 313 |
| Khan, 2015 | - Interviews - Supporting Household Activities for Health, Assets, and Revenue Dinajpur Survey | NR | Direct costs  Cost per capita of health care last week, round 1:1  Cost per capita of health care last week, round 2: 1  Cost per capita of health care last week, round 3: 1 |
| Khaing, 2015 | - Interviews - Pre-tested, structured questionnaires | - Public, private, pharmacy - Secondary and tertiary | Direct costs  Mean cost of health care last year  Q1: 134; Q2: 107; Q3: 213; Q4: 448; Q5: 3,409 |
| Wingfield, 2016 | - Interviews - Locally validated questionnaire | - Public | Mean cost of tuberculosis episode  Direct costs  T1: 805; T2: 772; T3: 946  Indirect costs: 972  Total costs: 1868 |
| Wingfield, 2016 | - Interviews - Locally validated questionnaire | - Public | Mean cost of tuberculosis episode  Direct costs  T1: 805; T2: 772; T3: 946  Indirect costs: 972  Total costs: 1868 |
| Wang, 2016 | - Survey, interviews - Urban Household Survey and Rural Household Survey of the National Bureau of Statistics of China Survey Office | NR | Direct costs  Annual cost per adult equivalent per household reported 2011  Q1: 97; Q2: 152; Q3: 217; Q4: 376; Q5: 449  Annual cost per adult equivalent per household reported 2012  Q1: 89; Q2: 150; Q3: 200; Q4: 293; Q5: 605 |
| Chen 2016 | - Interviews, medical charts - Structured questionnaire | - Public - Primary, secondary and tertiary | Mean cost of chronic conditions last year  Direct costs  Q1: 4,230; Q2: 3,429; Q3: 4,089; Q4: 3,828; Q5: 3,683  Indirect costs  Q1: 1,002; Q2: 614; Q3:748; Q4: 776; Q5: 318  Total costs  Q1: 5,532; Q2: 4,043; Q3:4,837; Q4:4,604; Q5: 4,001 |
| Mishra, 2016 | - Semi-structured interviews - Questionnaire pre-tested in slum | - Public; private; Informal; pharmacy; clinics of non-governmental organisations | Direct costs  Mean cost of child care, last illness episode: 51 |
| Kien, 2016 | - Interviews | NR | Direct costs  Mean cost of chronic conditions last year, slum  Q1: 789; Q2: 845; Q3: 906; Q4: 1,001; Q5: 1,695  Mean cost of chronic conditions last year, non-slum  Q1: 1,510; Q2: 1,674; Q3: 2,243; Q4: 2,014; Q5: 1,531 |
| Khalid, 2016 | - Survey, interviews - Household Income Expenditure Survey | NR | Direct costs  Mean cost of health care last year, 1999  D1: 2,269; D2: 4,500  Mean cost of health care last year, 2002  D1: 2,038; D2: 3,377  Mean cost of health care last year, 2005  D1: 1,949; D2: 2,883  Mean cost of health care last year, 2006  D1: 2,016; D2: 3,070  Mean cost of health care last year, 2011  D1: 2,113; D2: 2,097  Mean cost of health care last year, 2011  D1: 1,386; D2: 1,374 |
| Jeyashree, 2017 | - Survey, interviews - National Sample Survey | - Public and private | Direct costs  Median cost of hospitalization last year, public sector  Q1: 105; Q2: ;110; Q3: 181; Q4: 245; Q5: 399  Median cost of hospitalization last year, private sector  Q1: 757; Q2: 566; Q3: 503; Q4: 762; Q5: 990 |
| Hendrix, 2017 | - Standard case report, daily review of medical charts and interviews | - Public - Secondary and tertiary | Total costs  Mean cost of diarrhoea episode  Q1: 96; Q2: 72; Q3: 67; Q4: 62; Q5: 48 |
| Sahu, 2017 | - Survey, interviews | - Public, private, home | Direct costs  Mean cost of normal delivery: 82  Mean cost of caesarean: 542  Mean cost of normal delivery in public sector: 75  Mean cost of normal delivery in private sector: 207  Mean cost of caesarean in public sector: 220  Mean cost of caesarean in private sector: 954  Mean cost of delivery in public sector: 100  Mean cost of delivery in private sector: 705  Mean cost of home delivery: 48 |
| Khan, 2017 | - Survey, interviews - National Household Income and Expenditure Survey | NR | Direct costs  Mean cost of health care last year  Q1: 185; Q2: 206; Q3: 318; Q4: 383; Q5: 372 |
| Xu, 2018 | - Survey, interviews - National Health Services Survey | NR | Only CHE reported |
| Sharma, 2018 | - Interview, available medical records and bills - Locally validated questionnaire | - Public and private - Tertiary and home delivery | Indirect costs  Mean cost of home delivery: 20  Mean cost of government delivery: 123  Mean cost of private delivery: 236 |
| Mukama, 2018 | - Interviews - Structured questionnaire | - Public, private, informal (traditional healer), self-medication - Primary, secondary and tertiary | Mean direct cost of child care last year: 80  Mean indirect cost of child care last year: 14 |
| Ranjan, 2018 | - National survey, interviews - Social Consumption: Health survey | - Public and private | Direct costs  Median cost of hospitalization last year, public sector with insurance  Q1: 119; Q2: 101; Q3: 270; Q4: 205; Q5: 164  Median cost of hospitalization last year, public sector and no insurance  Q1: 182; Q2: 345; Q3: 371; Q4: 1,065; Q5: 833  Median cost of hospitalization last year, private sector with insurance:  Q1: 826; Q2: 1,063; Q3: 1,111; Q4: 1,176; Q5: 1,635  Median cost of hospitalization last year, private sector and no insurance  Q1: 1,102; Q2: 1,270; Q3: 1,495; Q4: 2,295; Q5: 2,506 |
| Cascaes, 2018 | - National survey, interviews - Pesquisas de Orcamentos Familiares | - Private - Primary | Direct costs  Per capita expenditure with health care  Q1: 160; Q2: 161; Q3: 281; Q4: 322; Q5: 844 |
| Kusuma, 2018 | - Interviews - Pretested, interview administered questionnaire | - Public and private clinics and hospitals, unqualified practitioners | Direct costs  Mean cost of health care last year  Q1: 663; Q2: 515; Q3: 666; Q4: 527; Q5: 576  Mean cost outpatient care last year  Q1: 91; Q2: 78; Q3: 96; Q4: 88; Q5: 105  Mean cost of inpatient care last year  Q1: 451; Q2: 440; Q3: 365; Q4: 378; Q5: 343 |
| Pandey, 2018 | - Survey, interviews - National Sample Survey Organization: survey on health care of 1995/96 (52nd round); survey on morbidity and healthcare of 2004 (60th round); and survey on social consumption: health of 2014 (71st round) | - Public and private | Direct costs  Mean annual cost inpatient last year, elderly, 1995  Q1: 112; Q2: 139; Q3: 303; Q4: 434; Q5: 1,095  Mean annual cost inpatient last year, elderly, 2004  Q1: 602; Q2: 1,038; Q3: 1,090; Q4: 1,596; Q5:3,359  Mean annual cost inpatient last year, elderly, 2014  Q1: 450; Q2: 518; Q3: 864; Q4: 1,095; Q5: 2,322  Mean annual cost inpatient last year, young, 1995  Q1: 96; Q2: 135; Q3: 201; Q4: 283; Q5: 733  Mean annual cost inpatient last year, young, 1995  Q1: 307; Q2: 406; Q3: 458; Q4: 577; Q5: 733  Mean annual cost inpatient last year, young, 1995  Q1: 446; Q2: 550; Q3: 581; Q4: 717; Q5: 1,342 |
| Enweronu-Laryea, 2018 | - Interviews - Patient resource-use measurement questionnaire | - Public - Tertiary | Direct costs  Mean cost of neonatal services  Q1: 430; Q2: 463; Q3: 683 |
| Sepehri, 2019 | - National survey, interviews - Vietnam Household Living Standards Survey | - Public and private - Primary, secondary and tertiary | Only CHE reported |
| Ntambue, 2019 | - Structured questionnaire, medical records, expert opinion, document review, semi-structured interviews - Semi-structured interview | - Public, private, religious, para-statal commercial, private, non-religious - Primary, secondary and tertiary | Only CHE reported |
| Banerjee, 2019 | - Interviews | NR | Direct costs  Mean cost of health care last year: 62 |
| Attia-Konan, 2019 | - National survey, interview - 2015 Standard Households Living Survey | - Public and private curative or preventive health services, over-the-counter medications and use of traditional healer services | Direct costs  Mean cost of chronic conditions last year  Q1: 145; Q2: 207; Q3: 241; Q4: 262; Q5: 387 |
| Acharya, 2019 | - Interviews - Global Aging and Adult Health, questions on Health Care Utilization tool | - Government hospital, Private hospital, health post, community hospitals, army camp, ayurvedic - Secondary and tertiary | Direct costs  Median costs of last outpatient visit  T1: 0; T2: 2; T3: 2 |
| Bose, 2019 | - National survey, interviews - National Sample Survey 71st round unit level data on Social Consumption: Health (2014) | - Public and private - Secondary and tertiary | Direct costs  Mean cost of inpatient care last year  Q1: 380; Q2: 581 ; Q3: 753; Q4: 1,351  Mean cost of outpatient care last 15 days, public  Q1: 12; Q2:26; Q3: 29; Q4: 33  Mean cost of outpatient care last 15 days, private  Q1: 36; Q2: 59; Q3: 51; Q4: 68 |
| Ma, 2019 | - Interviews - China Family Panel Studies (2010, 2012, 2014, and 2016) | NR | Only CHE reported |
| Leng, 2019 | - Interviews | - Secondary and tertiary | Direct costs  Mean cost of health care last year  Q1: 7,707; Q2: 7,254; Q3: 7,608; Q4: 7,785; Q5: 9,024 |
| Muniyandi, 2020 | - Interviews - The World Health Organization handbook for TB patient cost surveys | - Public, private, NGO - Primary, secondary and tertiary | Only CHE reported |
| Poornima, 2020 | - Interviews - The tool to estimate patient's costs. Tuberculosis Coalition for Technical Assistance | - Public, private, Informal, pharmacy | Total costs  T1: 47; T2: 408; T3: 593 |
| Adams, 2020 | - Interviews - Bangladesh Urban Health Survey Bangladesh Demographic and Health Survey,  Household Income and Expenditure Survey | - Drug shops, government hospitals, private hospitals or clinics, doctors chambers - Primary and secondary | Direct costs  Mean cost of chronic conditions last year in Tongi  Q1: 930; Q2: 1,313; Q3: 916; Q4: 1,234; Q5: 1,994  Mean cost of chronic conditions last year in Sylhet  Q1: 441; Q2: 555; Q3: 645; Q4: 865; Q5: 721 |
| Swetha, 2020 | - Interview, log book - Pretested semi structured questionnaire, log book | - Private clinics, government, nursing home, over the counter medicine, alternative system | Only CHE reported |

NR= Not reported; CHE= catastrophic health expenditures; Q1 to Q5= wealth quintile (from poorest to richest); Q1 to Q4= wealth quartile (from poorest to richest); T1 to T3= wealth tertial (from poorest to richest); D1 to D2= wealth decile (poor, non-poor or below, above poverty line)

Table S6. Characteristics of the Cost-analysis

| Reference | Direct Costs | | Indirect costs | Health insurance | Catastrophic Health Expenditure | |
| --- | --- | --- | --- | --- | --- | --- |
|  | Medical | Non-medical |  |  | Method | Threshold^1^ |
| Das, 2010 | √ | √ | √ |  |  |  |
| Barros, 2011 | √ | √ |  | √ | Annual household consumption and annual household income | 40% |
| Lopera, 2011 | √ | √ |  | √ |  |  |
| Garcia, 2012 | √ |  |  | √ |  |  |
| Saini, 2012 | √ | √ |  |  |  |  |
| Skordis-Worrall, 2012 | √ | √ | √ |  | Monthly household Expenditure | 40% |
| Bhojani, 2012 | √ | √ |  |  | Monthly household income | 5%, 10%, 15% and 20% |
| Kumar, 2012 | √ | √ |  | √ | Annual household income | 25% |
| Weraphong, 2013 | √ | √ |  | √ | Monthly household capacity to pay | 10% |
| Sarker, 2013 | √ | √ | √ |  |  |  |
| Sakdapolrak, 2013 | √ | √ |  |  |  |  |
| Misra, 2013 | √ | √ | √ |  | Monthly household capacity to pay | 10% |
| Rahman, 2013 | √ | √ |  |  | Monthly household capacity to pay | 40% |
| Patel, 2013 | √ | √ | √ |  |  |  |
| Seeberg, 2014 | √ |  |  | √ | Monthly household income | 10% |
| Ilesanmi, 2014 | √ | √ |  | √ | Monthly non-food expenditure | 40% |
| Wingfield, 2014 | √ | √ | √ |  | Annual household income | 20% |
| Chenge, 2014 | √ | √ |  |  |  |  |
| Navneet, 2014 | √ | √ |  |  |  |  |
| Saito, 2014 | √ | √ |  |  | Monthly household consumption | 10% |
| Rehman, 2014 | √ | √ |  |  |  |  |
| Prabhakaran, 2014 | √ | √ |  |  |  |  |
| Tripathi, 2014 | √ | √ | √ |  | Monthly household consumption | 10% |
| Chandra, 2014 | √ | √ | √ |  |  |  |
| Joe, 2015 | √ |  |  | √ |  |  |
| da Silva, 2015 | √ | √ |  | √ | Monthly household income | 5%, 10%, 15% |
| Patle, 2015 | √ | √ |  |  |  |  |
| Putri, 2015 | √ |  |  | √ | Annual household capacity to pay | 40% |
| Thakare, 2015 | √ | √ | √ |  | Annual non-food expenditure | 5%, 10%, 15%, 20% and 40% |
| Buigut, 2015 | √ | √ |  |  | Monthly household capacity to pay | 5%, 10%, 15%, 20% and 30% |
| Davari, 2015 | √ | √ |  | √ | Annual household capacity to pay | 40% |
| Loganathan, 2015 | √ | √ | √ |  | Monthly household income | 10% |
| Khan, 2015 | √ | √ |  |  | Monthly household consumption | 15% |
| Khaing, 2015 | √ |  |  |  | Annual household non-food expenditure | 40% |
| Wingfield, 2016 | √ | √ | √ |  | Annual household income | 20% |
| Wang, 2016 | √ |  |  |  |  |  |
| Chen 2016 | √ | √ | √ | √ |  |  |
| Mishra, 2016 | √ | √ |  |  |  |  |
| Kien, 2016 | √ | √ |  |  | Monthly household capacity to pay | 40% |
| Khalid, 2016 | √ |  |  |  |  |  |
| Jeyashree, 2017 | √ | √ |  |  |  |  |
| Hendrix, 2017 | √ | √ | √ |  |  |  |
| Sahu, 2017 | √ | √ |  |  | Monthly household income | 10% and 40% |
| Khan, 2017 | √ | √ |  |  | Annual household consumption and non-food expenditure | 5%, 10%, 15%, 25%, 40% |
| Xu, 2018 | √ | √ |  | √ | Annual household capacity to pay | 40% |
| Sharma, 2018 | √ | √ | √ |  | Annual household income | 10% |
| Mukama, 2018 | √ | √ | √ |  |  |  |
| Ranjan, 2018 | √ | √ |  | √ | Annual household consumption | 10% and 25% |
| Cascaes, 2018 | √ |  |  | √ | Annual household income | 5%, 10%, 20% |
| Kusuma, 2018 | NR | NR | NR | √ |  |  |
| Pandey, 2018 | √ | √ |  |  |  |  |
| Enweronu-Laryea, 2018 | √ | √ | √ | √ |  |  |
| Sepehri, 2019 | √ | √ |  |  | Monthly household expenditure | 40%, 30%, and 20% |
| Ntambue, 2019 | √ |  |  |  | Monthly household capacity to pay | 40% |
| Banerjee, 2019 | √ |  |  |  |  |  |
| Attia-Konan, 2019 | √ |  |  | √ |  |  |
| Acharya, 2019 | √ | √ |  | √ |  |  |
| Bose, 2019 | √ | √ |  |  |  |  |
| Ma, 2019 |  |  |  | √ | Annual household capacity to pay | 40% |
| Leng, 2019 | √ |  |  | √ | Monthly household income | 40% |
| Muniyandi, 2020 | √ | √ | √ |  | Annual household income | 20% |
| Poornima, 2020 | √ | √ | √ |  | Monthly household income | 10% |
| Adams, 2020 | √ | √ |  |  | Annual non-food expenditure | 10%, 15%, 25% |
| Swetha, 2020 | √ | √ | √ |  | Annual household income | 10% |

CHE= Catastrophic health expenditure

^1^ Threshold related to the method and time period indicated in the previous column (methods applied to calculate CHE)

Table S7. Quality assessment

| **Reference** | **Q1** | **Q2** | **Q3** | **Q4** | **Q5** | **Q6** | **Q7** | **Q8** | **Q9** | **Q10** | **Q11** | **Q12** | **Q13** | **Q14** | **Q15** | **Q16** | **Score (%)** | **Category^1^** |
| --- | --- | --- | --- | --- | --- | --- | --- | --- | --- | --- | --- | --- | --- | --- | --- | --- | --- | --- |
| Joe, 2015 | √ | √ | √ | √ |  |  | √ |  | * | * |  |  | √ |  |  |  | 43 | Poor |
| Jeyashree, 2017 | √ | √ | √ | √ |  | √ | √ | √ | * | * |  |  |  | √ |  |  | 57 | Poor |
| Ilesanmi, 2014 | √ | √ | √ | √ |  | √ | √ | √ | * | √ | √ | √ |  | √ |  | √ | 80 | Moderate |
| Hendrix, 2017 | √ | √ | √ |  | √ | √ |  | √ | √ | * | √ | √ |  |  | √ |  | 67 | Poor |
| Garcia, 2012 | √ | √ |  |  |  |  |  | √ | * | * | √ |  |  |  |  |  | 29 | Poor |
| Xu, 2018 | √ | √ | √ | √ |  | √ | √ |  | * | √ |  |  |  | √ |  | √ | 60 | Poor |
| Wingfield, 2016 | √ | √ | √ |  |  | √ | √ | √ | √ | √ | √ | √ | √ | √ | √ | √ | 88 | Good |
| Wingfield, 2014 | √ | √ | √ | √ |  | √ | √ | √ | √ | √ | √ | √ | √ | √ | √ | √ | 94 | Good |
| Weraphong, 2013 | √ | √ |  | √ | √ |  | √ | √ |  | √ |  | √ |  | √ | √ |  | 63 | Poor |
| Wang, 2016 | √ | √ | √ | √ |  |  |  | √ | * | * |  | √ |  | √ |  | √ | 57 | Poor |
| Sharma, 2018 | √ | √ | √ | √ | √ |  | √ | √ |  | √ |  | √ | √ | √ | √ | √ | 81 | Moderate |
| Sepehri, 2019 | √ | √ | √ | √ |  |  | √ | √ | * | √ |  |  | √ | √ |  |  | 60 | Poor |
| Seeberg, 2013 | √ | √ | √ | √ |  | √ | √ |  | * | √ |  | √ | √ | √ |  |  | 67 | Poor |
| Sarker, 2013 | √ | √ | √ | √ |  | √ | √ | √ | √ | * | √ | √ | √ | √ | √ | √ | 93 | Good |
| Sakdapolrak, 2013 | √ | √ | √ |  |  | √ | √ | √ | √ | √ | √ | √ | √ |  |  |  | 69 | Poor |
| Das, 2010 | √ | √ | √ | √ |  | √ | √ | √ |  | * |  | √ | √ | √ | √ | √ | 80 | Moderate |
| da Silva, 2015 | √ | √ | √ |  |  | √ |  | √ | * | √ | √ |  | √ |  | √ | √ | 67 | Poor |
| Chenge, 2014 | √ | √ | √ | √ | √ | √ | √ | √ | * | * | √ | √ | √ | √ |  |  | 86 | Good |
| Chen 2016 | √ | √ | √ |  |  | √ | √ | √ | √ | * |  | √ | √ | √ | √ | √ | 80 | Moderate |
| Ntambue, 2019 | √ | √ | √ |  |  | √ | √ | √ | * | √ | √ | √ | √ | √ | √ | √ | 87 | Good |
| Navneet, 2014 | √ | √ | √ | √ |  |  |  | √ |  | * |  | √ | √ | √ |  |  | 53 | Poor |
| Muniyandi, 2020 | √ | √ | √ |  | √ | √ |  | √ | √ | √ | √ | √ | √ | √ | √ | √ | 88 | Good |
| Mukama, 2018 | √ | √ | √ | √ | √ | √ |  | √ |  | * | √ | √ | √ | √ | √ |  | 80 | Moderate |
| Misra, 2013 | √ | √ | √ | √ | √ |  | √ | √ |  | √ |  | √ | √ |  |  | √ | 69 | Poor |
| Mishra, 2016 | √ | √ | √ |  |  | √ |  | √ | * | * |  | √ | √ | √ |  | √ | 64 | Poor |
| Barros, 2011 | √ | √ | √ | √ | √ |  | √ |  | * | √ |  |  | √ | √ |  | √ | 67 | Poor |
| Banerjee, 2019 | √ | √ | √ | √ |  |  | √ | √ | * | * |  | √ | √ | √ |  |  | 64 | Poor |
| Attia-Konan, 2019 | √ | √ | √ | √ |  |  |  | √ | * | * |  | √ | √ |  |  | √ | 57 | Poor |
| Saito, 2014 | √ | √ |  | √ | √ | √ | √ |  | * | √ | √ | √ | √ | √ | √ | √ | 87 | Good |
| Saini, 2012 | √ |  | √ | √ | √ | √ |  |  | * | * |  | √ | √ | √ |  | √ | 64 | Poor |
| Sahu, 2017 | √ | √ | √ | √ |  | √ | √ | √ | * | √ |  | √ | √ | √ |  | √ | 80 | Moderate |
| Rehman, 2014 |  | √ | √ | √ | √ | √ |  | √ | * | * |  | √ | √ | √ | √ | √ | 79 | Moderate |
| Ranjan, 2018 | √ | √ | √ | √ |  |  | √ | √ | * | √ |  |  | √ |  |  | √ | 60 | Poor |
| Rahman, 2013 | √ | √ | √ | √ |  |  | √ | √ | * | √ |  | √ | √ | √ | √ | √ | 80 | Moderate |
| Prabhakaran, 2014 | √ | √ |  | √ |  |  | √ | √ | * | * |  | √ | √ |  |  |  | 50 | Poor |
| Poornima, 2020 | √ | √ | √ |  |  | √ | √ | √ |  | * | √ | √ | √ | √ |  | √ | 73 | Poor |
| Patle, 2015 | √ |  | √ | √ | √ |  |  |  | * | * |  | √ | √ | √ | √ | √ | 64 | Poor |
| Patel, 2013 | √ | √ | √ |  |  | √ |  | √ | √ | * |  | √ |  |  |  |  | 47 | Poor |
| Putri, 2015 | √ | √ | √ | √ | √ |  |  | √ | * | √ |  | √ | √ | √ |  |  | 67 | Poor |
| Adams, 2020 | √ | √ | √ | √ |  | √ | √ | √ | * | √ | √ | √ |  | √ |  |  | 73 | Poor |
| Acharya, 2019 | √ | √ | √ | √ | √ | √ | √ | √ | * | * |  | √ | √ | √ |  | √ | 86 | Good |
| Tripathi, 2014 | √ | √ |  |  |  |  |  | √ | * | √ |  | √ | √ |  |  | √ | 47 | Poor |
| Thakare, 2015 | √ | √ | √ |  | √ | √ | √ | √ | √ |  |  | √ | √ |  | √ | √ | 75 | Moderate |
| Swetha, 2020 | √ | √ | √ | √ | √ | √ |  | √ |  | √ |  | √ | √ | √ |  |  | 69 | Poor |
| Skordis-Worrall, 2012 | √ | √ | √ | √ |  | √ | √ | √ |  | √ | √ | √ | √ | √ | √ | √ | 88 | Good |
| Chandra, 2014 | √ | √ | √ | √ |  |  | √ | √ |  | * |  | √ |  | √ |  |  | 53 | Poor |
| Cascaes, 2018 | √ | √ | √ | √ |  |  | √ | √ | * | √ |  | √ |  |  |  |  | 53 | Poor |
| Buigut, 2015 | √ | √ | √ | √ |  | √ | √ | √ | * | √ | √ | √ |  | √ |  |  | 73 | Poor |
| Bose, 2019 | √ | √ |  | √ |  |  | √ | √ | * | * |  |  |  |  |  |  | 36 | Poor |
| Bhojani, 2012 | √ | √ | √ |  |  | √ |  | √ | * | √ |  | √ | √ | √ |  | √ | 67 | Poor |
| Davari, 2015 | √ | √ |  | √ |  |  | √ |  | * | √ | √ |  | √ | √ | √ | √ | 67 | Poor |
| Ma, 2019 | √ | √ |  |  |  |  | √ | √ | * | √ | √ | √ | √ |  |  | √ | 60 | Poor |
| Lopera, 2011 | √ | √ | √ | √ | √ | √ | √ | √ | * | * | √ | √ | √ | √ |  | √ | 93 | Good |
| Loganathan, 2015 | √ | √ | √ |  | √ | √ |  | √ | √ | √ | √ | √ |  | √ |  | √ | 69 | Poor |
| Leng, 2019 | √ | √ | √ | √ | √ |  |  | √ | * | √ | √ | √ |  | √ |  | √ | 67 | Poor |
| Kusuma, 2018 | √ | √ | √ | √ | √ |  |  |  | * | * |  | √ | √ | √ | √ | √ | 71 | Poor |
| Kumar, 2012 | √ | √ | √ |  |  | √ | √ | √ | * | √ | √ | √ | √ |  | √ | √ | 80 | Moderate |
| Kien, 2016 | √ | √ | √ | √ | √ |  | √ |  | * | √ |  | √ | √ | √ | √ | √ | 80 | Moderate |
| Khan, 2017 | √ | √ | √ | √ |  |  | √ |  | * | √ |  |  | √ |  |  | √ | 53 | Poor |
| Khan, 2015 | √ | √ | √ | √ | √ |  |  |  | * | √ |  | √ | √ |  | √ | √ | 67 | Poor |
| Khalid, 2016 | √ | √ |  | √ |  |  |  |  | * | * |  | √ |  |  | √ |  | 36 | Poor |
| Khaing, 2015 | √ | √ | √ | √ |  |  |  |  | * | √ |  |  | √ | √ |  |  | 53 | Poor |
| Pandey, 2018 | √ | √ |  |  |  | √ | √ | √ | * | * | √ |  | √ | √ |  | √ | 64 | Poor |
| Enweronu-Laryea, 2018 | √ | √ | √ | √ |  | √ |  | √ | √ | * | √ | √ | √ | √ | √ | √ | 80 | Moderate |

* Not applicable; **^1^** Categories: good: >85%, fair: 75% - 84%, poor: <75%

Q1: Was the research question or objective in this paper clearly stated?

Q2: Was the period of data collection reported? CHEERS

Q3: Was the study setting clearly described? CHEERS

Q4: Was the sampling method described and adequate? NIH

Q5: Was a sample size justification, power description, or variance and effect estimates provided? NIH

Q6: Were inclusion and exclusion criteria described and adequate? NIH

Q7: Was the methodology applied to calculate income level described and adequate? *New question*

Q8: Were types of costs, out of pocket expenditures clearly defined? *New question*

Q9: When included, was the method used for valuing productivity losses explained and justified? CHEERS

Q10: When included, was the method used for estimating catastrophic costs explained? CHEERS

Q11: Was the method for adjusting unit costs to the reporting year and performing currency conversion explained? CHEERS

Q12: Is there a clear description of patient interview procedures given? TBCA

Q13: Was the participation rate of eligible persons at least 50%? NIH

Q14: Was the study population clearly described? NIH

Q15: Was denominator or refusals, or incomplete forms adequately reported? NIH

Q16: Was the result analysed and reported for all included participants? CHEERS
